# Cognitive Networks for Knowledge Modeling: A Gentle Introduction for Data‐ and Cognitive Scientists

**DOI:** 10.1002/wcs.70026

**Published:** 2026-03-10

**Authors:** Edith Haim, Massimo Stella

**Affiliations:** ^1^ CogNosco Lab, Department of Psychology and Cognitive Science University of Trento Rovereto Trentino Italy

**Keywords:** cognitive modeling, cognitive networks, datasets for network building, network science, quantitative analysis

## Abstract

In this paper, we introduce the reader to the field of cognitive network science, that is, the application of network science methods to study human cognition and knowledge structures. Cognitive networks are representations of associative knowledge between concepts in a cognitive system apt at acquiring, storing, processing and producing language, that is, the mental lexicon. In a cognitive network, nodes represent concepts with links expressing relations, such as semantic, syntactic, phonological and visual connections, for example, “canine” and “dog” (nodes) linked by “being synonyms” (link). Hence, cognitive networks represent associative knowledge in mathematical, measurable and quantifiable ways. Can such structure be used to gain insights over cognitive phenomena? We explore this research question by reviewing recent, pioneering key applications and limitations of cognitive networks across visual, auditory, and semantic language processing tasks, either in healthy or clinical populations. We also review applications of cognitive networks modeling language acquisition, reconstructing text content and assessing creativity or personality traits in individuals. Our paper also gently introduces the reader to mathematical notations, definitions and measures about single‐layer and multiplex networks as well as hypergraphs. Last but not least, across phonological, semantic and syntactic networks, we guide the reader through relevant psychological frameworks, datasets and software packages that might all aid current and future cognitive network scientists.

This article is categorized under:

Psychology > Memory
Psychology > Theory and Methods
Linguistics > Cognitive

## Graphical abstract

Cognitive network science helps organize associative knowledge—that is, the connections between concepts. These connections play a key role in cognitive processes such as language understanding and context interpretation, even though they are not obvious in language use. For example, we do not see syntactic links, as depicted in the figure, between words in a written or spoken text. Further information can be highlighted visually in cognitive representations, such as the emotional valence of words (here indicated with the colors blue for positive, red for negative, gray for neutral and purple for a connection between positive and negative concepts). Giving structure to knowledge via cognitive networks represents a new frontier. This gentle primer offers a clear overview and introduces tools for cognitive scientists and psychologists interested in exploring cognitive representations.

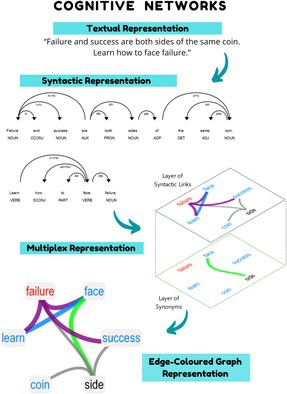

## 1 Introduction

Network science is the study of how elements are connected and how structure influences behavior, dynamics, or information flow across a system (Newman 2018). Within the broader field of network science (which can also be applied to areas like biology or social systems), the subfield of cognitive network science uses network‐based methods to study cognition (Siew et al. 2019). This paper introduces core ideas and tools from network science with a focus on language‐based networks as used in cognitive science, particularly to model conceptual and linguistic representations. Our approach to network science for cognitive researchers uses a representational framework. This representational perspective is based on the idea that our minds use internal representations to understand and process information. Such internal representations are mental models that stand for real‐world information. For instance, a real‐world tree may be represented by the word “tree” and a mental image of a prototypical tree (Markman and Dietrich 2000). These mental representations form a complex system where different levels, such as sounds and meanings, work together to convey knowledge, meaning, and emotions (Tulving 1972; Aitchison 2012; Zock 2020; Hills and Kenett 2021).

The complexity of cognitive systems comes from the idea that “more is different”: Combining ideas with specific features can create novel cognitive entities with distinctive characteristics. For example, combining “clause” (proposition) with “Santa” (saint) gives rise to the unique idea of “Santa Clause.” This kind of complexity is not limited to meaning or emotion, but also extends to many other aspects of knowledge. For instance, we have the ability to remember and combine small sound units, called phonemes, to form words (Vitevitch 2008). We then can build sentences by stacking words together. Multiple sentences can be joined into longer texts or narratives, which express ideas, viewpoints, and emotions. All these elements are highly interconnected in the mind.

All this information is stored and processed across several cognitive mechanisms, either sequentially or in parallel (Aitchison 2012; Dóczi 2019). These idealized linguistic forms, such as words and sentences, are structured representations of knowledge within human cognition. Other examples include mental images or conceptual frameworks, for example, visualizing a red apple when thinking about the concept “apple” (Ciaglia et al. 2023).

If concepts can be assembled together to build up our knowledge, then we need models to help explain, measure, and explore how these connections form (Dóczi 2019; Hills and Kenett 2021). These models should also show how the structure of associations between concepts relates to other cognitive phenomena, such as memory, learning, or language use (Stella et al. 2017).

To explore these ideas, cognitive modeling introduced the metaphor of a mental lexicon (Collins and Loftus 1975; Aitchison 2012). Contrary to its name, this is not a mere dictionary but rather a complex and dynamic system, made up of multiple interacting elements. The mental lexicon mostly includes semantic memory, acting as a repository for linguistic knowledge about concept meanings (Sizemore et al. 2018; Kenett et al. 2018; Kumar 2021), alongside other subsystems handling phonological knowledge (Vitevitch 2008), visual cues (Kennington and Schlangen 2015), and more (Dóczi and Kormos 2015; Zock 2020). In this lexicon, mental representations can share similar features, forming a “cobweb” of interactions influencing knowledge acquisition, storage, processing, and production (Steyvers and Tenenbaum 2005; Aitchison 2012). This analogy aligns with the concept of a complex network (Collins and Loftus 1975) but with a unique twist: links in the mental lexicon are not directly visible. Unlike physical networks, like brain circuits or transportation systems, we cannot observe the structure of the mental lexicon directly. This makes the structure of the mental lexicon inaccessible for direct, exact reproduction in a lab setting. For example, even though we can scan a brain or map out conceptual associations, we cannot directly see whether someone stores “happy” and “joyful” as synonyms in their mind. To investigate this, researchers must rely on indirect evidence, such as word associations or response times.

Consequently, the structure of the mental lexicon has to be indirectly investigated through cognitive tasks. A key issue with these tasks is that they represent linguistic data without providing insights into the cognitive structure of associative knowledge behind the data itself. For instance, the data coming from a fluency task (see Section 2.6) remains a sequence of words, with no explicit information about relationships between them. Hence, accessing only data from cognitive tasks still misses information about how concepts are organized within human cognition. This is where network models of cognition come into play. They serve as indirect tools or proxies to represent how concepts might be connected in the mental lexicon, even though we cannot measure that structure directly (Kenett and Hills 2021). Despite this distinction, the field of cognitive network science has grown over the years as a data‐centric field, employing multidisciplinary techniques from cognitive science, psychology, social science, mathematics, physics, statistics, and computer science (Hills et al. 2009; Kenett et al. 2018; Siew et al. 2019; Siew 2020; Stella 2022a).

This paper integrates excellent pre‐existing reviews (Siew et al. 2019; Siew 2020) with a special focus on two goals:

- outlining the most recent advancements and cognitive interpretations in the field focusing on network science methods and literature relevant for cognitive scientists, and
- introducing the latest large‐scale datasets and coding packages that can support researchers in using cognitive networks as models for understanding human cognition and behavior.

## 2 Basic Definitions

Network science examines patterns of connections among elements and investigates how these links affect the organization and functioning of systems. The broad area of network science includes many types of networks, such as social networks (e.g., groups of friends) (Mitchell 1974), biological networks (e.g., molecular interaction networks) (Alm and Arkin 2003), and financial networks (e.g., flow of finances between institutions) (Gale and Kariv 2007). While this paper introduces the reader to foundational concepts and tools from general network science, we focus only on a specific subset of networks: language‐based cognitive networks. In these models, nodes represent linguistic or conceptual units (such as words or meanings), and edges capture cognitive or linguistic relationships (such as semantic similarity, phonological overlap or syntactic dependencies).

### 2.1 Language‐Based Cognitive Networks

Let us start by defining in simple terms what a cognitive network is:

Definition 1 Cognitive network.

A cognitive network is a data‐informed model explicating associations (links) between cognitive representations of concepts (nodes).

Being data‐informed implies that cognitive networks draw their insights and structure from empirical data and psychological models (Siew et al. 2019; Stella 2022a). Thus, they reflect real‐world patterns and interactions with underlying theoretical assumptions and psychological constructs. The phrase “explicating associations” underscores the networks' ability to account for one or several connections or relationships present between concepts in the mental lexicon, for example, phonological similarities (Vitevitch 2008), memory recall patterns (De Deyne et al. 2013), syntactic dependencies between words (Stella 2020a) and so on.

Notice that the terms “words” and “concepts” are distinct. Words are linguistic units (sound forms or written tokens), while concepts are cognitive representations of ideas in the human mind (Vigliocco et al. 2018). For instance, the concept *apple* may be encoded verbally as “a‐p‐p‐l‐e,” but it also involves sensory experiences (e.g., visual form, taste, smell), personal memories, and cultural associations. The word form is typically arbitrary (De Saussure 1998), as evidenced by different translations like German “Apfel,” Italian “mela,” or Vietnamese “táo.” Thus, words can be understood as verbal proxies for concepts, which themselves are rich, multimodal constructs. Therefore, in this manuscript we refer to “concepts” when discussing the underlying cognitive representations of meaning, and to “words” when referring to the linguistic or phonological form. However, this distinction is not always clear‐cut in practice, as most experimental data and linguistic usage access concepts through their verbal proxies. Thus, both terms are often used interchangeably in the literature.

When we speak of “cognitive representations of concepts,” we refer broadly to the mental features associated with linguistic items, including semantic, phonological, emotional, visual, syntactic, and sensorimotor dimensions (Dóczi and Kormos 2015). For example, a single concept such as “bicycle” can be represented across multiple modalities. Semantically, “bicycle” is linked to related concepts such as “vehicle,” “transport,” or “ride” (Dóczi 2019). Phonologically, it shares sound similarities with words like “icicle” or “tricycle,” forming associations based on overlapping phoneme sequences (Vitevitch 2008). Morphologically, the underlying root “cycle” might connect “bicycle” to other morphologically related words such as “unicycle” or “motorcycle.” Syntactically, it functions as a noun that can appear in constructions like “The child rides the bicycle,” establishing syntactic relationships with verbs and modifiers (Stella et al. 2022). On the affective dimension, “bicycle” may evoke positive emotional associations such as “freedom,” “play,” or “childhood joy” (Semeraro et al. 2022). Visually, the concept may trigger mental imagery of a two‐wheeled frame with handlebars and pedals, while sensorimotor associations could include the feeling of balancing, pedaling, or steering (Ciaglia et al. 2023). Thus, a single concept can serve as a crucial node within a cognitive network, connecting across multiple types of associations within the mental lexicon (Martinčić‐Ipšić et al. 2016; Stella et al. 2018; Siew et al. 2019; Castro and Siew 2020; Levy et al. 2021; Stella 2022a).

In this paper, we focus specifically on language‐based cognitive networks. These networks are built from language data and are used to explore cognitive functions like learning, comprehension, and retrieval of knowledge. These networks can take the form of single‐layer or multilayer structures. Importantly, language‐based cognitive networks are not artificial neural networks (Fatima et al. 2021). The latter are interconnected computational units, that is, artificial neurons, which can integrate or modify signals over time. Thus, they possess a distinctive computational power, such as solving logic problems (Fatima et al. 2021). In contrast, the networks in this paper are representational rather than computational models. They explicate the structure of associative knowledge without implementing dynamic or mechanistic processes.

We also distinguish our focus from other types of network models that are commonly used in psychology and neuroscience. One such example is psychometric networks, which model statistical correlations among questionnaire items (Golino and Epskamp 2017). Psychometric networks capture relationships between numerical responses, not between conceptual representations. Furthermore, their methods for constructing links are based on statistical techniques rather than cognitive associations (Golino et al. 2020). That said, some pioneering approaches (Stanghellini et al. 2023) are entwining the structures of cognitive and psychometric networks, merging the semantic content of items in psychometric questionnaires with numerical responses. Still, due to the more statistical nature of correlations in psychometric networks, they will not be further discussed in this paper.

We also do not cover brain networks, which depict physical or functional connectivity between regions of the brain (Beaty et al. 2018). While brain networks can be used to predict cognitive traits such as creativity, they encode neural‐level relationships between brain circuits rather than associations between mental representations. As such, they lie outside the scope of this paper.

### 2.2 Simple Mathematical Definitions: Vertices and Edges

Before diving into more complex structures, we introduce two basic definitions of standard network theory (Newman 2018).

Definition 2 Vertex (node).

A vertex (node) is an element of the vertex set

$$V=\left\{v_{1},v_{2},\ldots ,v_{n}\right\}$$

where each $v_{i}$ represents a single linguistic or conceptual unit in the mental lexicon. The term “node” is preferred in network science, whereas “vertex” is more commonly used in graph theory.

Definition 3 Edge (link).

An edge (link) is an element of the edge set

$$E=\left\{e_{1},e_{2},\ldots ,e_{n}\right\}$$

where each $e_{k}=\left(v_{i},v_{j}\right)$ encodes a relationship or association between two vertices $v_{i}$ and $v_{j}$. The term “link” is commonly used in network science, whereas “edge” is preferred in graph theory.

**FIGURE 1:**
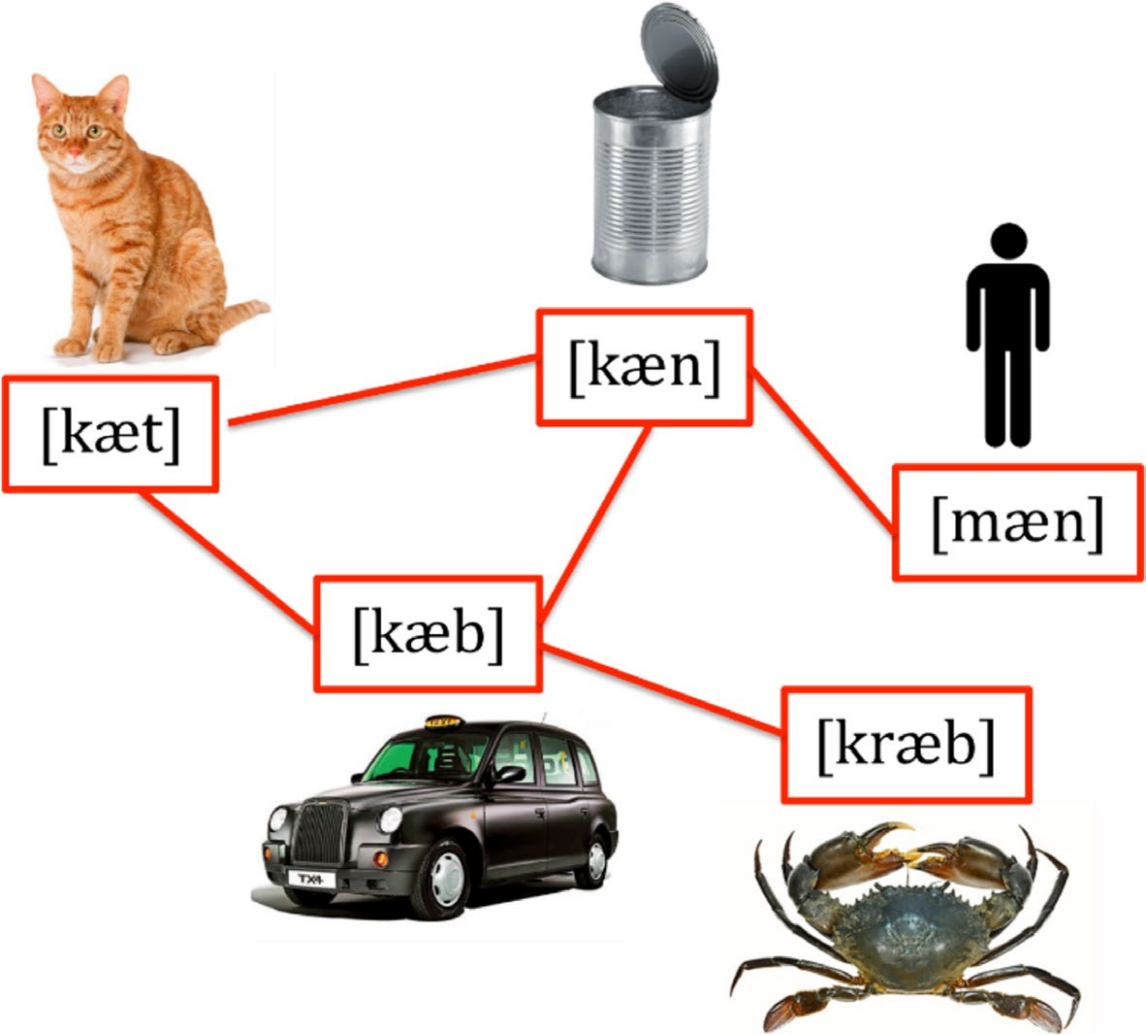
A cognitive network where nodes represent phonological transcriptions of English words written using the International Phonetic Alphabet (IPA; see also Turnbull 2021). The links between transcriptions indicate phonological similarities (i.e., two words differing for the addition, substitution or deletion of one phoneme only and thus sounding similar to each other).

A single‐layer network can then be represented as a couple of sets $\left(V,E\right)$, which includes the vertex set (consisting of the concepts) and the edge set *E* (consisting of the links between concepts) (Siew et al. 2019; Stella 2022a). For example, in a phonological network as depicted in Figure 1, the vertex set and edge set would be described as

$$V=\left\{\mathrm{cat},\mathrm{can},\mathrm{cab},\mathrm{man},\text{crab}\right\}$$

and

$$E=\left\{\left(\mathrm{cat},\mathrm{cab}\right),\left(\mathrm{can},\mathrm{cab}\right),\left(\mathrm{can},\mathrm{cat}\right),\left(\mathrm{man},\mathrm{can}\right),\left(\mathrm{cab},\text{crab}\right)\right\}$$

where each edge links words based on their phonological transcriptions, differing by a single phoneme that is added, substituted, or deleted (Turnbull 2021; Vitevitch 2008).

For instance, considering the vertex set of Figure 1, “cab” can be changed into “crab” by adding an/r/ sound, while substituting the/c/ in “can” for /m/ results in “man.” These examples showcase how slight phonological modifications can result in a series of words with interconnected phonological similarities, that is, sounding similar with each other. Figure 1 is a single‐layer cognitive network because it encapsulates links of only a single type of association (phonological similarities) (Stella et al. 2017).

Edges in a network can be undirected (symmetric, bidirectional relation) or directed (asymmetric, hierarchical relation). In undirected associations, the relationship between two concepts, represented as the link (*i*, *j*), is symmetrical, meaning it is equivalent to the reverse order (*j*, *i*). This symmetry is denoted as *i*‐*j*, indicating that the connection between the two concepts is bidirectional. For example, considering the synonyms “land” and “earth,” the undirected link “land”‐“earth” conveys that both terms are conceptually related with no directionality or hierarchy between them. Importantly, in undirected links, the order in which concepts are connected is irrelevant, emphasizing the mutual and interchangeable nature of these types of associations.

Instead, in directed associations the order of concepts is relevant (Stella et al. 2018). A directed link from concept *A* to concept *B* (A→B) generally indicates that *A* is associated with *B* in a specific, directional way. The meaning of this direction depends on the type of relationship being modeled. It could represent causality (*A* causes *B*), information flow (from *A* to *B*), or hierarchy (*A* is a more general category than *B*). For example, consider the sentence “Birds are a broader category of doves.” We can model this as a directed link bird→dove, where the direction of the arrow reflects that “bird” is a hypernym (a broader category) of “dove.” In other contexts, however, the direction may represent the opposite: in word association tasks, participants might respond to the stimulus “dove” with “bird.” In this case, the link would be modeled as dove→bird, reflecting an association from “dove” leading to “bird.” Hence, bird→dove could be represented as $\left(\text{bird},\text{dove}\right)$. Instead, dove→bird, or equivalently $\left(\text{dove},\text{bird}\right)$, would encode a different type of relationship than the reversed directed link.

Networks where all links are undirected are called undirected networks (Newman 2018). Networks with only directed links are called directed networks. Mixed networks combining directed and undirected connections can be treated as directed by representing each undirected link as a pair of reciprocal directed edges (Newman 2018). For instance, the relationship “good”‐“positive” can be split into “good” → “positive” and the reciprocating connection “positive” → “good.”

### 2.3 Single‐Layer, Multilayer and Multiplex Lexical Networks

**FIGURE 2:**
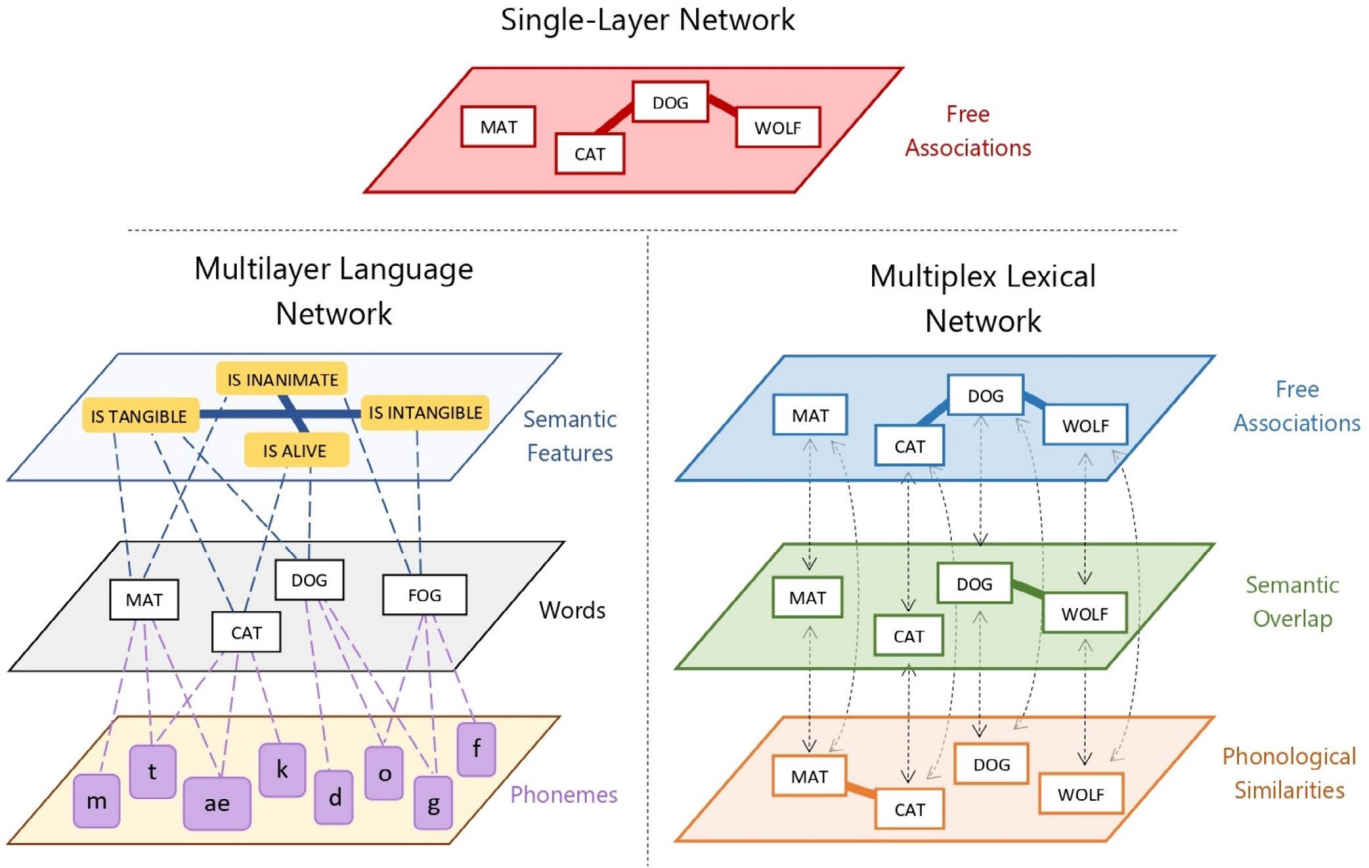
Network types with different numbers and types of layers. *Top*: Single‐layer network containing only one type of relationship. In this case it is built connecting free associations. *Left*: Multilayer language network capturing how distinct semantic features and phonemes are mapped onto a set of words. Each layer contains a different set of nodes. *Right*: Multiplex lexical network featuring associative, semantic, and phonological relationships. Each layer contains the same set of nodes, indicating different relationships between them.

Extensive psycholinguistic research has shown that the same set of concepts can be related through multiple types of associations (Fay and Cutler 1977; Aitchison 2012; Abrams and Davis 2016). These relationships can span different cognitive and linguistic domains, such as semantic similarity, phonological overlap, syntactic dependency, and free association. A single‐layer network captures only one type of relationship at a time. For instance, Figure 2 (top) illustrates a single‐layer network built from free association data, where “cat,” “dog,” and “wolf” are connected based on them belonging to the category of animals. Free association is a classic psycholinguistic method in which participants are asked to respond with the first word that comes to mind when presented with a cue word (Nelson et al. 2004).

Alternatively, a single‐layer network using the same set of concepts could highlight semantic overlap as in “dog” overlapping semantically with “wolf,” that is, sharing certain characteristics. Another single‐layer network constructed of phonological information could relate “cat” to “mat” due to their phonological similarity.

Since single‐layer networks isolate one type of relationship, they provide a limited view of conceptual connections (Newman 2018). To reflect the multidimensional structure of language, researchers can use multilayer or multiplex networks. These allow different types of relationships (e.g., associative, semantic, phonological) to be modeled in parallel (De Domenico 2022; Stella et al. 2024). Figure 2 depicts an example of a single‐layer network and two network types featuring multiple layers: multilayer networks (left) and multiplex networks (right).

Multilayer networks are highly flexible, allowing each layer to contain different sets of nodes and interactions (Newman 2018). This makes multilayer networks well‐suited for systems where not all entities are involved in every type of relationship (Kivelä et al. 2014). For example, in a language network as depicted in Figure 2 (left), one layer might capture semantic information about concepts (e.g., dog → is alive), while another layer represents the phonemes within the phonological word form (e.g., dog → /d/, /o/, /g/). Not all phonemes or semantic features map exactly onto all concepts, thereby creating layers with potentially differing numbers of nodes and interactions (Stella et al. 2024).

In contrast, multiplex networks are a specific subtype of multilayer networks where the same set of nodes exist across all layers. Each set of concepts is mapped exactly onto the next layer, with each layer representing a distinct type of interaction among the same entities (Stella et al. 2017, 2018; Stella 2020b). This structure is especially useful when studying how different types of relationships coexist among a consistent set of elements. In the example depicted by Figure 2 (right), a multiplex lexical network could represent the same set of concepts (e.g., “mat,” “cat,” “dog,” “wolf”) and capture a different dimension of connectivity among them, such as free associations, semantic overlap or phonological similarities, among many other types. In general, in a multiplex lexical network, multiple edge‐lists $E_{1},\ldots ,E_{L}$ co‐exist, each one composing a distinct network layer with the same set of nodes (Stella et al. 2024).

Historically, the idea of different layers originated in social science contexts for mapping different types of social relationships among the same set of individuals (Newman 2018). In cognitive science, relationships of different sorts indicate distinct aspects of associative knowledge being simultaneously or sequentially used for acquiring, storing, and processing concepts (Hills et al. 2009; Citraro, De Deyne, et al. 2023). For instance, the phenomenon of malapropism (Fay and Cutler 1977) occurs when someone can access the phonological information relative to a given target word but then fails at activating the correct semantic information that would lead to using the target word in the correct context. An example of malapropism could be “I band but I don't break” where we use “band” instead of “bend.” The occurrence of malapropism in common language signifies that phonological and semantic layers are distinct but interact with each other during language processing and production (see also Dóczi 2019). This importance is further underlined by another phenomenon rising from the interactions between semantic and phonological aspects of the mental lexicon, that is, the so‐called “tip of the tongue” event (Abrams and Davis 2016). This takes place when someone can access the semantic features of a given target word but is unable to retrieve and produce the phonological instruction for naming the target. For instance, someone might be familiar with the concept of a crane (e.g., being a bird, having wings, flying) but ultimately being unable to say the name at the moment and rather use a common English expression: “I have it on the tip of my tongue.” Malapropisms and tip‐of‐the‐tongue events both underline how important it is to identify models that can encapsulate at the same time semantic and phonological relationships between concepts, like multiplex lexical networks.

**FIGURE 3:**
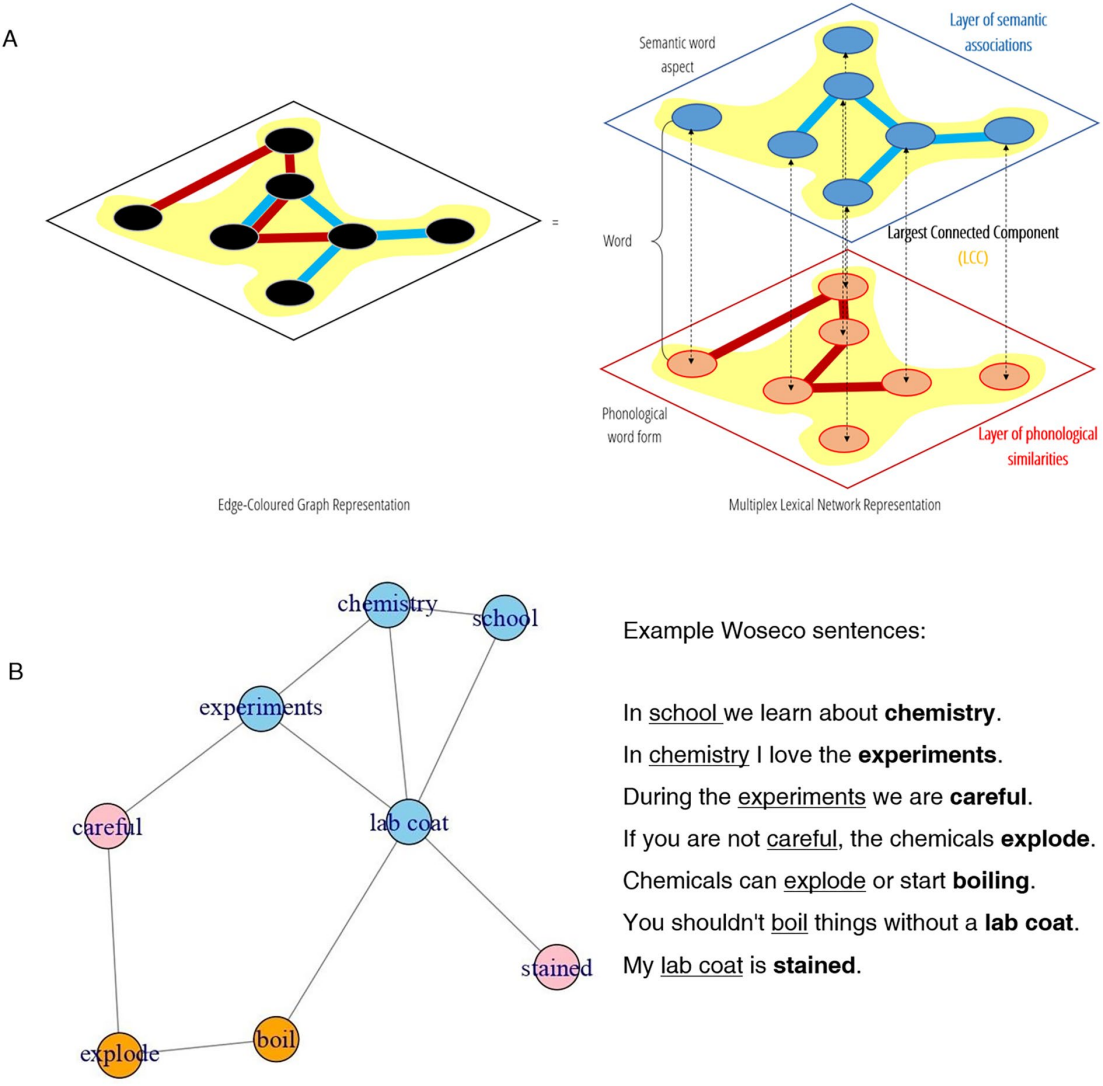
Examples of an edge‐colored graph and a feature‐rich graph. Panel A: A multiplex lexical network where nodes are connected through phonological (red) and semantic (cyan) associations. These networks can be visualized by either emphasizing their multi‐layer structure (right, multiplex representation), where all nodes are replicated across layers, or by collapsing layers together while preserving different colors (left, edge‐colored representation). The largest connected component of the network is highlighted in yellow. Panel B: A Woseco network visualized as a feature‐rich graph. The nodes are formed by words being repeated across the chain of sentences. The edges represent the syntactic relationships between the words within the text. Nodes are color‐coded to indicate their word category. Nouns are depicted in blue, verbs in orange and adjectives in pink. This figure was created using the *igraph* library in R.

Multiplex networks can exist with (Figure 3, right) or without (Figure 3, left) explicit links between different layers. Some multiplex networks include explicit links between layers to model interdependencies or interactions between different layers (see Figure 3, panel A right). For instance, in online social networks, users may have accounts on multiple platforms (e.g., Instagram, Facebook) and interlayer links can represent the association of accounts belonging to the same user (Khdir et al. 2024). In contrast, in many multiplex networks, once one identifies all the replicas of the same entity (e.g., all the replicas of “mat” across layers) there is no need to further specify direct unweighted links between layers, as shown in Figure 3, panel A left. In contrast, in many multiplex networks, there are no direct links between layers, as shown in Figure 3, panel A left. Instead of splitting the multiplex network into separate visually distinct layers and drawing connections between them (see Figure 3, right), this visual representation without explicit interlayer links collapses all layers together onto one level. The different types of relations between nodes as based on a different layer are indicated by links being drawn in different colors (see Figure 3, left). Therefore, multiplex networks without explicit interlayer links are also called “edge‐colored” graphs (Stella et al. 2017; De Domenico 2022). The left panel of Figure 3 panel A portrays an edge‐colored graph mixing phonological associations (highlighted in red) and semantic associations (highlighted in cyan).

In cognitive network science, modeling interactive or subsequent transitions between phonology and semantics poses a challenge (Vitevitch and Mullin 2021). As a result, most multiplex lexical networks in the current literature are presented as edge‐colored graphs (see Figure 3). In these edge‐colored graphs, different types of connections (e.g., phonological vs. semantic) are distinguished by colors (Stella et al. 2024). For instance, in Figure 3 panel A the semantic layer is portrayed in cyan while the phonological layer is depicted in red. This coding via colors allows for a clear visual distinction between the various types of associations within the multiplex network (Stella et al. 2017, 2018).

An alternative approach to visualizing multilayer networks is through feature‐rich graphs (Citraro, Vitevitch, et al. 2023), which allow for the integration of multiple attributes into a single representation. These graphs not only capture the connections between elements (e.g., words) but also information about their properties or roles in the network (Citraro, Vitevitch, et al. 2023; Interdonato et al. 2019). Feature‐rich graphs can contain various features such as lexical category (noun, verb, adjective), animacy (animate, inanimate), valence (positive, negative, neutral) or concreteness (concrete entities, abstract ideas). Figure 3, panel B depicts an exemplary feature‐rich graph using the feature of lexical category in a network constructed for the Woseco (Word‐sentence‐construction) task. In this task, participants create a chain of sentences that are linked to each other through a shared word repeated in both sentences (Haim, Lai, et al. 2024; Haim, Fischer, et al. 2024). For example, a Woseco text could go: “In school we learn about *chemistry*. In *chemistry* I love the experiments.” In Figure 3, panel B nodes represent words that are connected based on their sequence within the sentences, with edges reflecting the syntactic relationships between them. The nodes are color‐coded to indicate their grammatical properties such as word category (nouns are blue, verbs are orange and adjectives are pink). This feature‐rich design of a Woseco network highlights both the structural and grammatical aspects of the network.

The choice for features for such graphs depends on the research question. For instance, the lexical category (as used in Figure 3, panel B) may be used as a feature to investigate how lexical categories (noun, verb, adjective) are connected to each other in a network constructed from the Woseco task.

In the case of animacy, studies have found that animacy plays a role in memory, language production and processing, among others (Nairne et al. 2017; Vihman and Nelson 2019). For instance, animate targets are recalled more reliably than inanimate words (Nairne et al. 2017), which makes animacy a meaningful feature to include in network models of memory.

In relation to emotional valence, feature‐rich graphs (e.g., forma mentis networks) have been used to model negative emotional associations surrounding the concept of mathematics, reflecting how math anxiety is encoded in the cognitive networks of both humans and LLMs (Abramski et al. 2023; Stella 2022b).

Lastly, studies on vocabulary acquisition have found a processing advantage of concrete words over abstract ones, such that concrete words tend to be learned earlier and may serve as anchors in semantic networks for novel word learning (Ding et al. 2017; Kaushanskaya and Rechtzigel 2012).

Why use multiplex rather than simpler single‐layer networks? The combination of network layers might highlight phenomena that could not be observed in individual networks. For instance, in Siew and Vitevitch (2019) orthographic and phonological similarities highlighted facilitative effects in visual word recognition that were not observed in orthographic similarities or phonological similarities separately. Other examples include phenomena such as enhanced preferential acquisition (Stella et al. 2017) and improved lexical processing based on word distance in clinical populations (Castro and Stella 2019; Castro et al. 2020; Baker et al. 2023). These phenomena emerged only when semantic and phonological layers were combined in the multiplex structure: Multiplexity might open up novel perspectives when modeling semantic memory. For a more comprehensive review on the topic, we refer to Stella et al. (2022).

Edge‐colored graphs and multiplex lexical networks (Stella et al. 2018) can be used as synonyms whenever there are no explicit costs for transitioning between layers. Explicit costs for transitioning between layers could entail the effort, resources, or time required to navigate across these layers. For instance, in a cognitive network, moving from a semantic layer (containing conceptual information) to a phonological layer (representing the sound form of a word) may involve overcoming activation thresholds or activation costs (Siew and Vitevitch 2019; Stella et al. 2024). Consider spreading activation in this context. When a concept is activated (e.g., reading the word “apple” on a screen activates the conceptual representation), its associated activation spreads to its neighboring nodes and through the network. The spreading activation diminishes over distance and with each node it traverses (Collins and Loftus 1975; Koponen 2021). Similarly, inter‐layer transitions may also encounter diminishing activation energy or increased cognitive load, representing certain costs for traversing from one layer to the next. These costs may be higher for weak or distant connections and lower for strong connections between layers. For example, a weak semantic‐phonological coupling for an infrequent word might lead to difficulty in word retrieval, whereas stronger links could make transitioning more efficient (Koponen 2021; Siew 2019).

In cases where there are no explicit costs associated with transitions between layers, the connections may be highly efficient or seamlessly integrated, facilitating a free flow of activation without significant energy loss, delays or cognitive effort. For instance, a strong coupling of the semantic and phonological layers for highly frequent words might result in spreading activation from the conceptual representation (e.g., a concept is shown as a picture and shall be named) to its phonological form without significant costs. This scenario could occur when inter‐layer links are strong or tasks are highly automated, such as in fluent speech or reading, though empirical validation of such cases remains limited (Stella et al. 2018; Koponen 2021).

### 2.4 Simple Mathematical Definitions: Adjacency Matrices

Definition 4 Adjacency matrix.

An adjacency matrix is a square matrix that represents the connections between nodes in a network to describe the network topology.

Both single‐ and multiplex networks can be represented as matrices (Newman 2018; De Domenico 2022). Let us here consider the matrix for a single‐layer network (see Section 2.7 for a supra‐adjacency matrix of a multiplex network).

**FIGURE 4:**
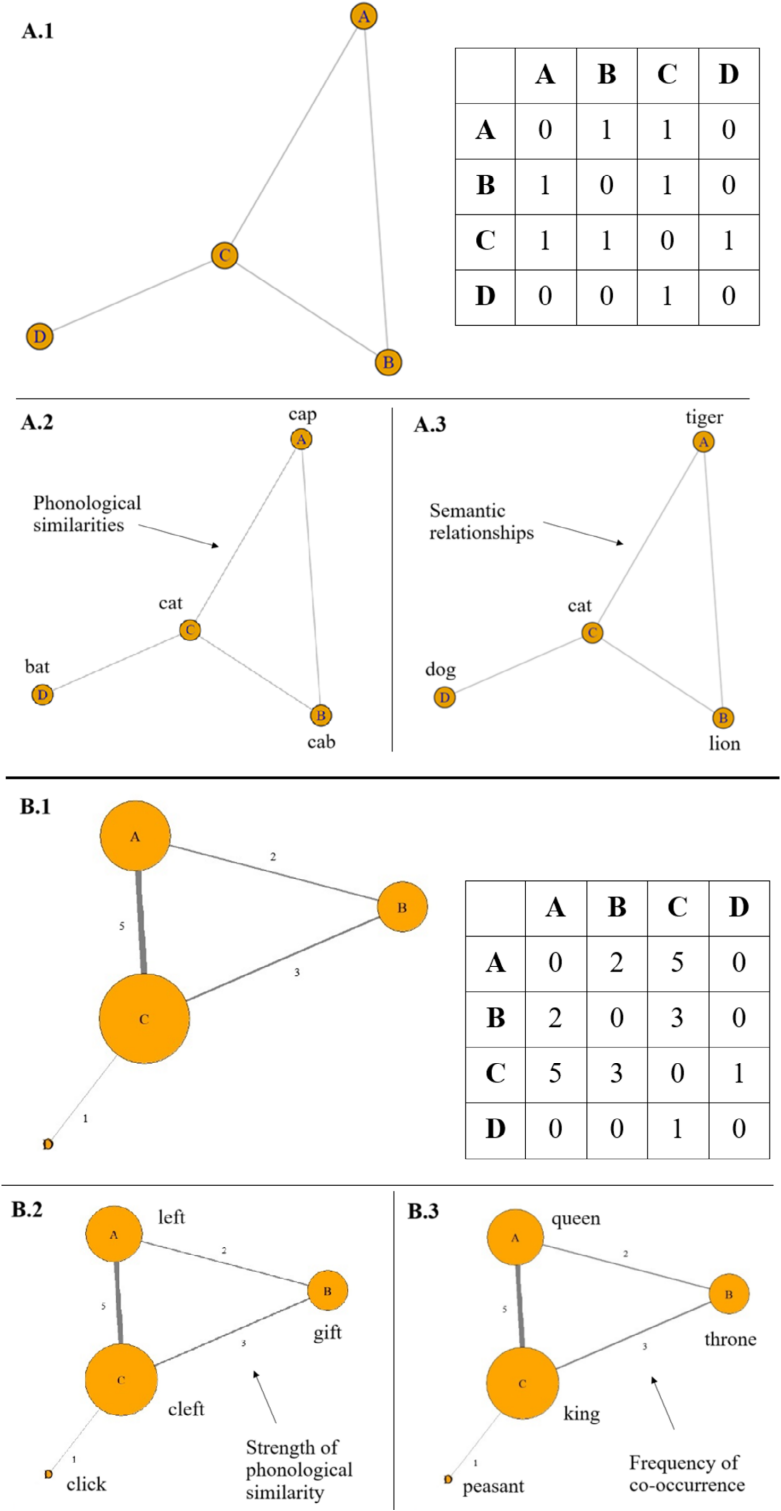
Panel A.1: Example of an undirected, unweighted single‐layer network (left) and its adjacency matrix (right). The existence or absence of connections between the entries (A–D) is indicated in the adjacency matrix as either 1 (link exists) or 0 (no link exists). A.2: Unweighted phonological network based on phonological similarities between the nodes. A.3: Unweighted semantic network based on semantic relationships between the nodes. These networks were created using the *igraph* library in R. Panel B.1: Example of an undirected, weighted single‐layer network (left) and its adjacency matrix (right). The strength of connections between the entries A, B, C and D is reflected in the adjacency matrix with higher numbers indicating greater weight of the link. B.2: Hypothetical example of a weighted phonological network, based on stronger or weaker phonological similarities. B.3: Hypothetical example of a weighted free association network, based on how frequently words co‐occur with each other. These networks were created using the *igraph* library in R.

For a single‐layer network with N nodes, the adjacency matrix S has the size N×N, where N=∣V∣ is the number of nodes in the network. Each element $s_{\mathrm{ij}}$ in the adjacency matrix indicates whether there is a link between node i and j (see Figure 4, panel A.1).

$$s_{\mathrm{ij}}=1\;\text{indicates the presence of}\;a\;\text{link between node}\;i\;\text{and}\;j$$

$$s_{\mathrm{ij}}=0\;\text{indicates the absence of}\;a\;\text{link between node}\;i\;\text{and}\;j$$

In undirected networks, the matrix is symmetric:

$$s_{\mathrm{ij}}=s_{\mathrm{ji}}\forall i,j\in V$$

This symmetry reflects that if node i is connected to node j, then node j is also connected to node i.

If a node is connected to itself, this is called a self‐loop and notated as follows:

$$s_{\mathrm{ii}}=1.$$

Otherwise, if there are no self‐loops, all diagonal elements from left to right are zero (see Figure 4, panel A.1). An undirected, unweighted network without self‐loops is also called a “simple network” (Newman 2018). Figure 4, panel A.1 depicts an example of an undirected, unweighted network with its respective adjacency matrix. To give a concrete example, consider the phonological network in Figure 4, panel A.2. The nodes “cat,” “cab,” and “cap” form a triangle in this simple network because they share phonological similarities with each other. However, the node “bat” only connects to “cat.” These connections are captured in the adjacency matrix (see panel A.1): node C representing “cat” shows connections to nodes A “cap,” B “cab,” and D “bat” which is indicated by 1s in the adjacency matrix. Since it has no self‐loop, there is no link from C to itself (indicated by 0). In contrast, node D “bat” only shares a link with node C “cat” that is marked by a 1. All other fields in the matrix from D to the remaining nodes are occupied by 0s. Similarly, Figure 4, panel A.3 shows a semantic network based on meaning‐related relationships between the entries. “Cat,” “lion,” and “tiger” (nodes C, B, and A) are all linked together, while “dog” (node D) is only connected to “cat.” The adjacency matrix for the semantic network in panel A.3 is the same as shown in panel A.1.

Weighted networks extend this representation by including a weight matrix W where each element $w_{\mathrm{ij}}$ represents the strength of the connection between *i* and *j*. Figure 4, panel B.1 shows an example of a weighted network and its adjacency matrix. Instead of a simple 1 indicating the presence of a link as in an unweighted network, the adjacency matrix of a weighted network reflects the strength of a link through smaller or larger numbers: larger numbers indicate a higher weight and stronger relationship between the nodes, while low numbers indicate smaller relationships. In case of bigger weight, the strength of the connection between two nodes is indicated through thicker links and nodes with higher weight are depicted larger.

In phonological networks, weights can encode the degree of phonetic similarity between words, highlighting how closely related they are in their sound form (Castro and Siew 2020). Figure 4, panel B.2 shows an example of a phonological weighted network where the nodes “cleft” and “left” share an edge with the strongest weight because they only differ by one phoneme. The edges from “cleft” and “left” to “gift” are slightly weaker since they have lower phonological similarity.

Alternatively, in semantic networks, weights may indicate the co‐occurrence of words in fluency experiment tasks, reflecting their associative relationship (Kenett et al. 2018). For instance, in Figure 4, panel B.3, the words “king” and “queen” are connected by an edge with higher weight because they co‐occur more often than each co‐occurs with “throne.” In contrast, the link between “peasant” and “king” has a very low weight due to their infrequent co‐occurrence.

Weights also allow researchers to capture asymmetries or varying intensities within cognitive systems. For instance, two words such as “degree” and “freedom” might co‐occur frequently in a context of statistics (as in “degrees of freedom”) but rarely in another more general context, resulting in different weights for their connections in different subsets. Weighted networks allow the identification of hubs with disproportionately strong connections. In contrast, simple unweighted networks (see Figure 4) treat all connections equally, which may hide meaningful variations in the underlying structure.

Notice that in this paper, we focus exclusively on positive weights, which typically reflect the strength or frequency of associative, co‐occurrence, or similarity‐based relationships (Castro and Siew 2020; Kenett et al. 2018). Some network models also use negative weights to represent inhibitory or contrastive relationships (MacLennan 2001). For instance, when modeling spreading activation, positive weights can indicate activatory edges (facilitating spreading activation), while negative weights indicate inhibitory edges (preventing the activation spreading along these edges) (Tsatsaronis et al. 2007). However, negative weights are primarily used in artificial neural networks for computational purposes (such as modeling inhibitory connections), whereas semantic networks in cognitive psychology typically use positive weights to model co‐occurrence or semantic relatedness. Since the networks discussed in the present paper are language‐based cognitive networks that focus on associative structures, we will not discuss negative weights in the remainder of this paper.

### 2.5 Simple Mathematical Definitions: Degree, Hubs, Power‐Laws, and Degree Distributions

Note: The definitions in this section focus on single‐layer networks. Generalizations of these definitions to multiplex networks can be found in Section 2.7.

Definition 5 Degree.

The degree $k_{i}$ of a node i in a network is the number of edges connected to it:

$$k_{i}=\sum_{j\;\epsilon \;V}s_{i,j}$$

where $s_{i,j}$ is the element of the adjacency matrix S, indicating the presence (Abrams and Davis 2016) or absence (0) of a connection between node i and j.

In simple single‐layer networks, summing over the i‐th column or row of matrix S provides the total number of links involving node i, indicating the network degree $k_{i}$. The network degree $k_{i}$ is a local network measure specific to node *i*: Degree counts only interactions that directly consider one node while neglecting the rest. In phonological networks, network degree has long been called phonological neighborhood density (PND), counting words which are phonologically similar to a given target (Vitevitch 2008; Turnbull 2021). Degree has been shown to influence lexical identification in confusability tasks and even short‐term retainment (cf. Vitevitch and Mullin 2021). For example, consider a node in a semantic network with a high degree, meaning it is connected to numerous other nodes. In a confusability task, that is, say whether a sequence of phonemes represents a real word, participants might find it challenging to quickly and accurately identify this node due to the abundance of interconnected associations, leading to potential confusion with closely related concepts. Conversely, in short‐term retention tasks, for example, memorize and reproduce words, a node with a high degree may be recalled more easily, as its rich connectivity aids in creating a robust and interconnected mental representation, facilitating easier retrieval from memory according to a spreading activation phenomenon (Vitevitch and Mullin 2021).

Definition 6 Hub.

A hub is a node whose degree is significantly higher than the network average:

$$k_{i}>\left\langle k\right\rangle$$

where ⟨*k*⟩ is the mean degree across all nodes in the network.

In practice, hubs are commonly identified as nodes falling in the upper percentiles of the degree distribution $p\left(K=k\right)$ (see Definition 7), typically the 95th percentile (Stella 2020b; De Domenico 2022).

Having a higher than average degree means a hub node is highly interconnected with other nodes and thus plays a central role in network connectivity and information flow. For example, in a semantic network, a node like “animal” may link to many specific concepts (e.g., “cat,” “dog,” “bird”), forming a hub. Hubs support rapid spreading activation and facilitate memory retrieval. Due to its importance for network connectivity, removing a hub node (e.g., a concept becomes inaccessible due to brain lesion) would significantly impact the network structure.

In semantic networks, hubs often represent pivotal concepts that play significant roles in information flow and cognitive processing (Stella 2020b). These hubs may correspond to central ideas or key semantic components that hold considerable influence over the overall network structure (De Arruda et al. 2017). Note that conceptual knowledge can, to some extent, emerge independently of language (Searle 2002). However, in this paper we focus on language‐based cognitive networks and will not discuss language‐independent cognition further.

By identifying and understanding these hubs in the context of semantic networks, cognitive network scientists can gain insights into the organizational principles of semantic networks, discerning the key nodes that shape information flow and contribute substantially to cognitive processes. For instance, hubs might be general‐level cues that mediate memory recollections of many other concepts (De Deyne et al. 2013). It was recently shown that hubs tend to be acquired earlier by normative English‐speaking toddlers, that is, children who follow typical language development timelines (Hills et al. 2009; Sizemore et al. 2018). In contrast the acquisition of semantic hubs is impaired in late learners (Beckage et al. 2011). Late learners are children who develop language skills more slowly than the majority of children, that is, normatively developing children, due to environmental or social factors (e.g., reduced language exposure) rather than any clinical or neurological causes (Beckage et al. 2011). For example, a typically developing English‐speaking toddler might readily acquire a semantic hub such as “apple” due to its common use and broad applicability in their everyday environment. Notice that social, cultural and geographic differences in everyday environments might change which concepts become early hubs. For instance, urban versus rural settings might shape a child's early vocabulary differently. In contrast to typically developing children, a late learner might struggle to acquire such foundational concepts, leading to difficulties in comprehending fundamental terms that serve as central nodes in their cognitive network which further interferes negatively with their ability to learn and connect other concepts in their semantic network (Beckage et al. 2011; Hills et al. 2009; Sizemore et al. 2018).

Definition 7 Degree distribution.

A degree distribution is the probability distribution of sampling a node with degree k:

$$p\left(K=k\right)=\frac{\text{number of nodes with degree}\;k}{\text{total number of nodes}}$$

It reflects how degrees are spread across the network.

Much attention has been devoted to characterizing a network based on the statistical distribution of its degrees (Steyvers and Tenenbaum 2005). Consider the probability $p\left(K=k\right)$ of sampling one node in a network and finding it has a degree equal to k. Sampling means considering one node at random by picking it among all available nodes in a given network structure. Thus, $p\left(K=k\right)$ is the fraction of nodes with that degree.

Definition 8 Power‐law.

A power law distribution describes the probability $p\left(K=k\right)$ of finding a node with degree k:

$$p\left(K=k\right)=z\cdot k^{-\alpha }$$

where z is a normalization constant and α is a power‐law exponent. In power‐law networks, most nodes may have low degree and a few nodes have very high degree (hubs).

If the probability distribution of finding a node with a specific degree follows a power‐law distribution, then the network is said to be a power‐law network. However, fitting power‐law distributions directly to $p\left(K=k\right)$ can be error‐prone, especially for nodes with infrequently high degrees (Alstott et al. 2014). A more robust method is to examine the cumulative degree distribution:

$$C\left(K\geq k\right)$$

which is the probability of finding a node with a degree equal to or higher than k.

Notice, however, that fitting a specific equation to an empirical degree distribution can be daunting not only in statistical terms but also for the interpretation of degree distributions. Power‐law degree distributions are interesting mostly because they indicate the presence of a few hub nodes thanks to their heavy‐tailed decay. The “tails” of a distribution refer to the extreme ends of the distribution, where the probability of observing values becomes progressively smaller. A distribution can have a “heavy tail” if the probability of extreme events, located in a tail, decreases more slowly than it would in a normal or exponential distribution. This implies that rare and high‐magnitude events, such as hubs, occur more frequently than expected in heavy‐tailed rather than non‐heavy‐tailed distributions (e.g., Gaussian distributions). Hence power‐law degree distributions in networks imply that a few nodes have exceptionally high degrees (Champagnat et al. 2013). However, this can be the case also in presence of other heavy‐tailed distributions, for example, log‐Gaussian distributions or stretched exponentials. Since from a cognitive perspective it remains unclear whether cognitive representations have a specific benefit in displaying power‐law degree distributions, the emphasis should be put not on detecting power‐lawness but rather in identifying hubs. For large values of k, the probability of finding a node with a high degree, that is, a hub, can be considerably higher for a heavy‐tailed than for an exponential distribution with the same average degree $\left\langle k\right\rangle$. For example, in a semantic network with a heavy‐tailed degree distribution, a small number of concepts—yet higher than in random graphs with exponential degree distributions—may serve as prominent hubs, forming numerous connections, while the majority of concepts have fewer connections (De Arruda et al. 2017; Newman 2018).

### 2.6 A Bit Less Simple Mathematical Definitions: Network Laplacians and Spreading Activation

Definition 9 Network laplacian.

A network Laplacian L of a simple, undirected graph is defined as:

L=D−S

where D is the degree matrix and S is the adjacency matrix of the graph.

**FIGURE 5:**
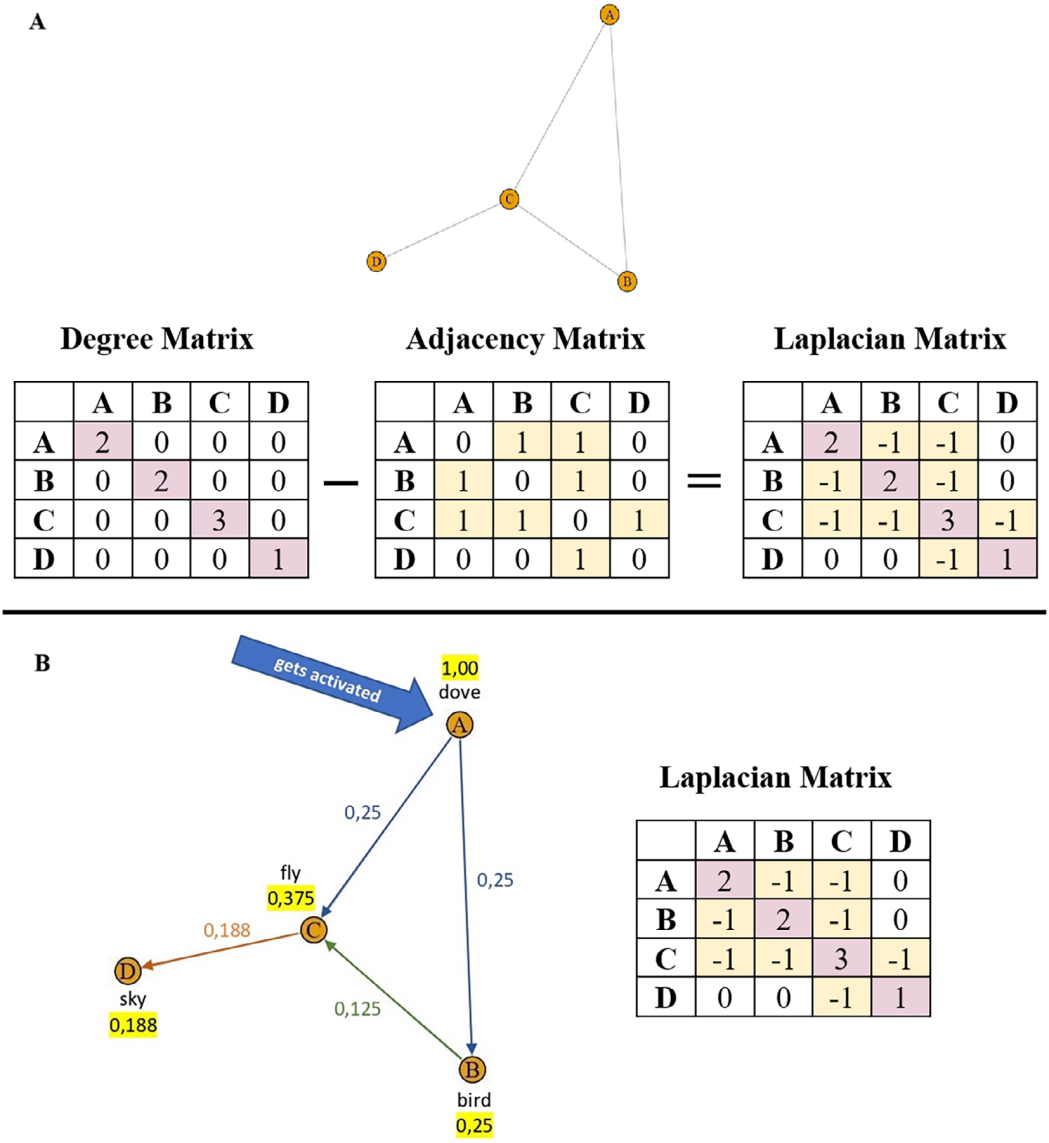
Example of a simple network with its Laplacian Matrix. Panel A: A simple network and its respective degree matrix, adjacency matrix and Laplacian matrix. The degree matrix captures the degree of each node on the main diagonal. The adjacency matrix highlights which nodes are connected with 1 if there is an edge between two nodes and 0 if there is no edge. The Laplacian matrix is calculated by subtracting the adjacency matrix from the degree matrix, indicating how easily the activation in the network can spread. This example network was created using the *igraph* library in R. Panel B: Spreading activation across a semantic network. In this example, nodes retain 50% of the activation they receive and spread the remaining 50% equally to their neighboring nodes. The strength of this spread of activation is noted next to the edges. The total activation received by a node is marked in yellow next to the node.

For any single‐layer simple network, the degree matrix D indicates the degree of a node on its main diagonal and 0s everywhere else. The adjacency matrix S of this network represents the actual connections a node has, indicated by 1 for existing edges and 0 if there is no edge between a given pair of nodes (see Figure 5A). Through S and D we can define a network Laplacian as L=D−S (Koponen 2021).

A Laplacian matrix encodes how each node in the network is connected to other nodes and provides a mathematical way of representing how activation can spread across a network. When a node is activated, the activation spreads through the edges and the Laplacian matrix quantifies how this spreading happens (Koponen 2021). Figure 5A provides a visual representation of a simple network consisting of four nodes and their respective degree matrix, adjacency matrix, and Laplacian matrix.

Let us discuss diffusive processes through a physical metaphor. Imagine a liquid with droplets flowing along links on a network structure relative to the Laplacian L. As portrayed in Figure 5A, the main diagonal of L comes from D (for simple networks) and it encodes information over the total flow that can accumulate across links surrounding each node, for example, one droplet per link. However, L contains also entries from the adjacency matrix S so each off‐diagonal entry in L indicates where droplets can arrive into or leave nodes when flowing through connections. Overall, it is expected that L regulates the diffusion of droplets across links and over nodes in a given network (Newman 2018).

The Laplacian L is also relevant to cognitive scientists modeling spreading activation in lexical retrieval (Siew 2019). Spreading activation was introduced by Collins and Loftus to model semantic priming (Collins and Loftus 1975). A prime node i receives an initial activation level $a_{i}\left(0\right)$. At the next time step, the activation $a_{i}\left(0\right)$ spreads uniformly across the $k_{i}$ links of i to its neighbors. When the activation spreads, at each time step the activation level from a given node—or part of it—is transmitted to its connected neighbors, influencing their subsequent activation levels. The signal can receive dampening or spread integrally based on factors such as the strength of connections between nodes. For instance, when flowing along connections, a part of the activation signal might get lost (uniform dampening). It might also be that some conceptual associations might be weaker and thus receive lower activation units, whereas other associations might be stronger and thus able to channel stronger activation signals (Collins and Loftus considered this latter case as “highways” able to withstand higher traffic loads, see Collins and Loftus 1975). Iteratively, the activation signals of all nodes with some activation levels have to spread across neighbors. The process continues iteratively until a final time step is reached, typically when the system achieves a stable state or a desired number of iterations have been reached (Collins and Loftus 1975; Castro and Siew 2020). Simulated activation levels on phonological networks were shown to replicate key findings in lexical processing (Vitevitch and Mullin 2021), indicating a crucial link between this model and some instances of knowledge processing in the mental lexicon.

For an example of spreading activation, consider activating the concept “dove” in the semantic network depicted in Figure 5B. Its activation will spread to related concepts, such as “bird,” “fly,” and “sky,” through the edges of the network. In Figure 5B this process of spreading activation is illustrated over successive timesteps with each timestep represented by a distinct color. At the first timestep (indicated by blue edges), activation flows from node A (“dove”) to nodes B and C (“bird” and “fly”). For this example we assume that each node retains 50% of its activation and distributes the remaining 50% evenly to its neighbors. This results in “bird” and “fly” receiving 25% of the initial activation. At the next timestep, the activation gets spread on further, for instance from “bird” to “fly”, contributing to an additional 12.5% of activation to node C. The strength of activation spreading is noted on the edges while the total activation received by each node is displayed at the node and marked in yellow. This highlights how activation spreads and accumulates across timesteps.

The Laplacian matrix helps to track how activation levels ripple through the network and how much activation remains after it has spread throughout the network. The off‐diagonal elements in the Laplacian matrix (highlighted in yellow) show the direct connections between nodes with negative values indicating the strength of potential activation flow. The diagonal elements (marked in pink) represent the node degrees and quantify the capacity of each node to spread activation to its neighbors. Nodes with higher degree possess greater potential to spread activation and have more influence on the overall activation dynamics within the network. For example, consider node A in Figure 5B. The Laplacian matrix indicates a degree of 2 for node A (diagonal entry $L_{\mathrm{AA}}=2$) and weights of −1 for the connections to B and C (off‐diagonal entries $L_{\mathrm{AB}}$ and $L_{\mathrm{AC}}$). When node A is activated, its energy is distributed proportionally to these links, meaning that activation flows equally to nodes B and C.

Importantly, network structure can influence the amount of activation a target node can receive, thus influencing its cognitive processing. Clustering, degree, and distance (see down below) can promote the concentration of activation over nodes related to the prime and thus suggest potential candidates for recollection (Siew 2019; Kumar et al. 2020). In cognitive networks representing the associative knowledge of students in the history of science, an appropriately normalized Laplacian was shown to capture nodes representing prominent scientists (Koponen 2021).

### 2.7 Generalizations to Multiplex Lexical Networks

**FIGURE 6:**
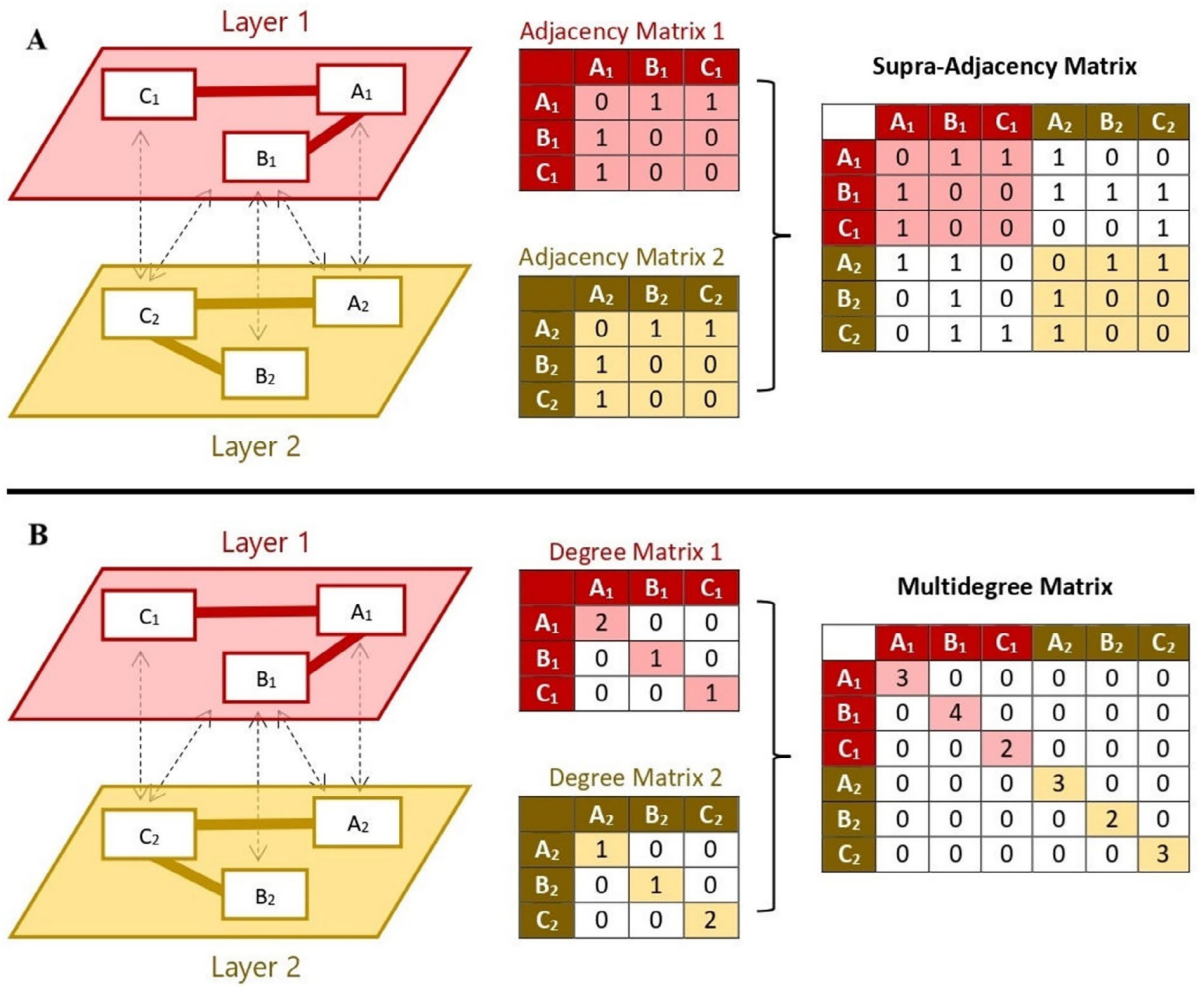
Panel A: Example of a supra‐adjacency matrix for a multiplex network consisting of two layers and three nodes. All nodes on the first layer are linked to their respective counterparts on the second layer. Notice that B_1_ also has intra‐layer links to A_2_ and C_2_. Panel B: Example of a multidegree matrix for a multiplex network consisting of two layers and three nodes. Node B_1_ has a multidegree of 4 since it has one edge within layer 1 and three intra‐layer links to A_2_, B_2_, and C_2_.

The above notions can all be extended to multilayer networks. In the case of this paper, let us focus on the simpler instance of multiplex networks (Battiston et al. 2017). A multiplex network is a type of network where the same set of nodes appear in multiple layers. The connections between nodes can differ from one layer to another, depending on the type of relationship captured by each layer. In the context of language‐based networks, a multiplex network might consist of a semantic and a phonological layer, where each repetition of a node represents the same word across both layers (Citraro et al. 2025). These multiple appearances are called replica nodes. Within each layer, nodes are connected based on a specific type of relationship (e.g., words linked by meaning in the semantic layer, or by sound similarity in the phonological layer). Furthermore, some nodes may also be connected across layers, representing how different kinds of information interact (see Figure 6A) (Citraro et al. 2025).

For a multiplex network, a supra‐adjacency matrix and a supra‐Laplacian matrix can be built as block matrices, with the diagonal elements being set to the adjacency (Laplacian) of individual layers and off‐diagonal elements being equal to diagonal matrices expressing inter‐layer relationships. An example of a supra‐adjacency matrix for a multiplex network consisting of two layers is depicted in Figure 6A.

For more details we refer the interested reader to De Domenico (2022). Applying multilayer or multiplex Laplacians to cognitive networks remains a scarcely explored research direction. Instead, extending the idea of degree to multiplex networks is relatively simpler. Summing degrees across layers, the multidegree $m_{i}$ of node i is defined as the following:

Definition 10 Multidegree.

In a multiplex network, the multidegree $m_{i}$ of node i is calculated by summing degrees over layers:

$$m_{i}=\sum_{\alpha }k_{i}^{\left(\alpha \right)}$$

where $k_{i}^{\left(\alpha \right)}$ is the degree of node i in layer α, for example, the number of cognitive links of layer α surrounding node i (Stella et al. 2017).

See Figure 6B for an example of a multidegree matrix derived from a 2‐layer multiplex network. In multiplex networks, α can enumerate the different replicas of a node across each layer. These multiple representations of a single node across different layers of the network are called replica nodes. Each replica node represents the same entity (e.g., a word) but in different layers, such as phonological, semantic and syntactic. Considering Figure 6B to be a semantic network, one word such as “cat” in node A could have two replica nodes: one on the phonological layer (A_1_) representing its sound structure as (kæt) and one on the semantic layer indicating its meaning as a small feline animal (A_2_) (Battiston et al. 2017).

In Figure 6B, the highest multidegree is 4 (of node B_1_). A node having a multidegree of 4 means that, when considering cognitive links across different layers, that node is connected to four distinct other nodes across all layers. The multidegree of a node might thus be different from the single‐layer degrees of the node's replicas. Multidegree can be used to define multiplex hubs: Nodes in the 95th percentile of the multidegree distribution. Stella (2020b) showed that a multiplex network representation of the mental lexicon with 16,000 English words over four semantic or phonological layers relied heavily on multidegree hubs to preserve connectivity (and thus a chance for activation spreading) among large portions of words. Removing these hubs made the network highly fragmented and disconnected, which hindered activation spreading from reaching several network regions, thus impairing word retrieval and comprehension.

In semantic multiplex lexical networks, multidegree is also called semantic richness, that is, the number of words syntactically or semantically related to a target (Aitchison 2012; Stella 2020a). Nodes with higher semantic richness correspond to more general concepts that may be used together with several other concepts or appear in a wider range of contexts (Semeraro et al. 2025). These words tend to be involved in more syntactic links within the network, which supports easier activation in a variety of linguistic contexts. Psychologically, this relates with the idea of priming, where activation of a concept facilitates the retrieval of related concepts (Siew 2019; Siew and Vitevitch 2019). Multidegree hubs can be especially effective during priming due to their high degree of connectivity across multiple layers. Such multidegree hubs might not only receive priming more easily because they are linked to a broad array of concepts across different layers, but they can also serve as relevant prime activators. Multidegree hubs' central role across multiple layers allows them to quickly spread activation, facilitating the retrieval of related concepts. For instance, when a multidegree hub like “cat” is activated, it can rapidly prime other related concepts in both the semantic layer (e.g., “dog” or “pet”) and the phonological layer (e.g., “bat” or “hat”) (Siew 2019). The investigation of these nodes in the framework of cognitive multiplex networks (Stella et al. 2024) remains an open yet interesting research area at the time of writing this paper.

### 2.8 Beyond Degree: Local and Global Clustering Coefficient

Whereas degree is a local feature of nodes, local clustering and closeness centrality capture meso‐scale and global aspects of network topology. Meso‐scale aspects refer to intermediate‐sized structures or patterns within the network, while global aspects describe the overall structure and efficiency of information flow across the entire network (Siew et al. 2019). Local clustering is a meso‐scale measure in that it characterizes a node based on links between its neighbors.

Definition 11 Local clustering coefficient.

The local clustering coefficient $C_{i}$ of a node i measures how interconnected the neighbors of node i are. It counts the ratio of neighbor‐pairs that are connected with each other:

$$C_{i}=\frac{|\left\{\left(k,l\right)\in E\;\text{for}\;k,l\in \partial_{i}\right\}|}{\frac{|\partial_{i}|\left(\left|\partial_{i}\right|-1\right)}{2}}$$

where $\partial_{i}$ is the set of neighbors of node i and the numerator counts how many links exist between those neighbors. A value of $C_{i}=1$ means that all neighbors of node i are connected to each other and form a perfect clique. $C_{i}=0$ indicates no interconnections among neighbors.

**FIGURE 7:**
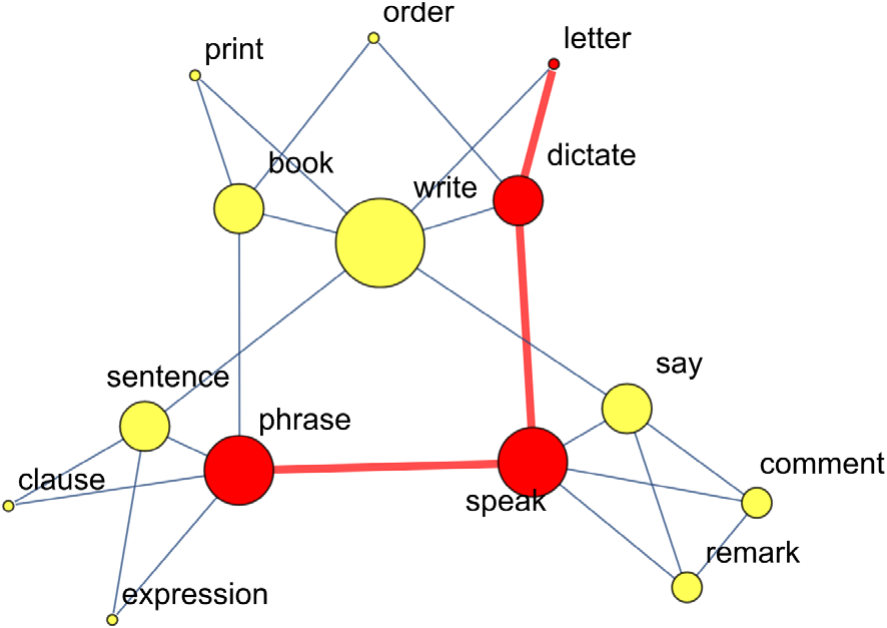
A cognitive network where nodes represent concepts and edges indicate undirected free associations (i.e., two words were mentioned as cue and recall in a free association task). The shortest path between “letter” and “phrase” is highlighted in red. Node size is proportional to their closeness centrality.

In other words, the local clustering coefficient measures a tendency for a node's neighbors to be linked with each other. $C_{i}$ ranges between 0 (no neighbors are linked) and 1 (all neighbors are linked) (Siew and Vitevitch 2019). Alternatively, $C_{i}$ measures how much the neighborhood of i resembles a complete network (where all nodes are adjacent to each other). In Figure 7, the concept “remark” has three neighbors that are all linked with each other, so $C_{\text{remark}}=1$.

High local clustering is associated with small‐worldness, a network property that combines high local clustering with short average path lengths between nodes (Newman 2018). In cognitive networks, this small‐world structure supports connectivity within clusters and rapid traversal across the network, similar to mechanisms underlying associative processes and memory retrieval (Siew 2020). In psychological terms, the property of small‐worldness reflects how efficiently information can be retrieved and connected in the human mind, supporting rapid access to related concepts (Newman 2018).

However, studies found that higher local clustering in phonological networks corresponded to degraded lexical identification, exemplified by the increased difficulty participants faced in accurately and swiftly recognizing and retrieving specific words during linguistic tasks (Chan and Vitevitch 2009). This can be understood through the lens of lexical competition, where densely clustered phonological representations lead to greater interference among similar words, slowing down retrieval. Activation for a target word accumulates along nodes in the neighborhood of node *i*, in formulas $\partial_{i}$, which makes it more difficult for i to stand out. Thus, in a phonological network with higher local clustering, participants were found to struggle to quickly distinguish between similar‐sounding words due to the increased interference and competition among closely interconnected or clustered phonological representations (Chan and Vitevitch 2009; Castro and Siew 2020).

In multiplex networks $C_{i}$ cannot be generalized in a single way, depending on how links from different layers are counted (De Domenico 2022). Siew and Vitevitch (2019) aggregated orthographic and phonological similarities and found that a higher $C_{i}$ corresponded to a facilitatory effect over spoken word recognition. In a multiplex network combining orthographic and phonological layers, a higher $C_{i}$ value might indicate a more interconnected relationship between written and spoken representations of words, leading to enhanced facilitation in recognizing and processing those words in spoken form (Siew and Vitevitch 2019).

### 2.9 Distance, Connectedness, and Closeness Centrality in Single‐Layer and Multiplex Networks

Definition 12 Network distance.

Network distance $d_{i,j}$ is the shortest number of links that must be traversed to connect a node i to node j in a network (Steyvers and Tenenbaum 2005):

$$d_{i,j}=\min\;\left(\text{length of}\;\mathrm{all}\;\text{possible paths from}\;i\;\text{to}\;j\right)$$

In Figure 7, “letter” and “phrase” are at distance $d_{\text{letter},\text{phrase}}=3$.

Definition 13 Connectedness and largest connected component.

Two nodes i and j are connected if there exists at least one path of finite, non‐zero length between them. In simple networks, the largest set of connected nodes is called the largest connected component (LCC).

In directed networks, one has to consider directionality for navigating the network structure from one node to another. Two nodes might have a sequence of links between them (i.e., a path) but directionality might impede getting out from one node to access another along the path. If this happens, those nodes would be said to be weakly connected (Newman 2018). Otherwise, if directionality would still provide access from one node to another along any given path, those nodes would be said to be strongly connected (Newman 2018).

In multiplex lexical networks, multiplex connectivity can exploit paths across all available layers such as connections between phonological and semantic layers within the same multiplex network (Stella et al. 2018). Consequently, multiplexity provides more shortcuts or opportunities for connecting any two nodes. Furthermore, replica nodes disconnected in one layer might be connected in another layer and thus, potentially, on the whole multiplex network too (see Figure 6B).

Cognitive network distance proves effective in capturing semantic relatedness and priming effects, as demonstrated by Kenett et al. (2017), Kumar et al. (2020), and Wulff, Aeschbach, et al. (2022). Kenett et al. (2017) found that in Hebrew, network distance in a free association network (where links indicate memory recall patterns) outperformed advanced models like latent semantic analysis when predicting data from a semantic relatedness judgment task. Kumar et al. (2020) replicated these findings in English and expanded the exploration of semantic distances to predict reaction times in priming tasks. They observed a linear increase in response latencies with network distance, revealing significant differences between prime‐target cases $d_{\mathrm{pt}}=1$ and $d_{\mathrm{pt}}=4$. For reviews on this topic see (Kumar 2021; Wulff, Aeschbach, et al. 2022).

Notably, network distance has also illuminated variations in sequential lexical retrieval across different populations, including those with distinct traits such as task‐quantified creativity levels (Stella and Kenett 2019). Assessments like the verbal fluency task serve as valuable tools for exploring creativity and its relation to semantic networks (see also Section 3.2). In a verbal fluency task individuals are asked to generate words within specific constraints, for example, naming as many animals as possible in 2 min (animal fluency task) (Goñi et al. 2011). Fluency tasks can be used in various forms, such as semantic fluency, where participants need to generate words belonging to a certain semantic category, or phonological fluency with the restriction that words need to begin with a particular letter. In semantic fluency tasks, individuals with higher creativity levels were found to produce recalls separated by longer network distances, on a multiplex network including almost 16k English words, compared to controls with lower creativity levels (Stella and Kenett 2019).

Whereas distance is a network property considering two nodes at a time, one could be interested also in assessing whether a single node is distant or close to all other nodes in a given connected component. In simple networks, this can be done by considering closeness centrality (Newman 2018).

Definition 14 Closeness centrality.

Closeness centrality measures how close a node is to all other nodes in its connected component. It reflects the efficiency with which a node can spread information through the network. If i belongs to a connected component with N nodes, then the closeness of i is calculated as:

$$c_{i}=\frac{1}{\left\langle d_{\mathrm{ij}}\right\rangle }=\frac{N-1}{\sum_{j}d_{\mathrm{ij}}}$$

where $d_{\mathrm{ij}}$ is the shortest path distance between nodes i and j, and where N−1 is a normalization factor excluding distances from self‐loops with $d_{\mathrm{ii}}=0$.

Closeness centrality is a global network feature because it considers the whole layout of shortest paths between a node and its whole connected component. A shorter average network distance is supposed to be advantageous for mental navigation because it allows for quicker and more efficient access to information, facilitating faster information retrieval and processing (Hills et al. 2012; Dubossarsky et al. 2017; Stella et al. 2018; Levy et al. 2021). In phonological networks, higher closeness centrality of words is associated with quicker reaction times in an auditory lexical decision task. In this task, participants must quickly determine whether a presented string of letters forms a real word or a non‐word. A quicker reaction time during this task highlights a beneficial role of shorter network distance, thus higher closeness, for facilitating faster lexical decision making (Goldstein and Vitevitch 2017).

In multiplex lexical networks, where there is no explicit cost for transitioning from one layer to another, network distance is defined as the shortest path length connecting any two nodes through links of any layer (Stella et al. 2018). For instance, if “cat” and “crab” are connected by a direct link in the semantic layer (since both are animals) and another direct link in the phonological layer (due to their phonological similarity), the network distance between “cat” and “crab” is considered as 1, regardless of the layers involved in the connection. Castro and Stella (2019) showed how higher multiplex closeness centrality predicted better performance in picture naming by people with aphasia disorders. As already mentioned above, multiplexity can provide additional shortcuts, lowering down the distance between concepts (Quispe et al. 2021). Levy and colleagues showed that the links provided by a phonological network present substantially more shortcuts to free association networks than random expectation; thus, enhanced cognitive advantage over word processing from combining free associations and phonological similarities (Levy et al. 2021). Words with higher multiplex closeness centrality were found to be learned earlier by normative learning toddlers (Stella et al. 2017). This latter finding is in agreement with the preferential acquisition hypothesis in cognitive psychology (Hills et al. 2009; Sizemore et al. 2018; Borovsky et al. 2021; Cox and Haebig 2022). The preferential acquisition hypothesis states that children exhibit a tendency to preferentially acquire information that is more central and well‐connected in their environment (cf. Cox and Haebig 2022). Essentially, the preferential acquisition hypothesis suggests that the ease of access and interconnectedness of a concept within one's cognitive structure both positively influence the likelihood of that concept being learned earlier in the developmental process (Hills et al. 2009). In this framework, high multiplex closeness centrality was shown to support early learning by making words more accessible within the whole multiplex network compared to individual layers (Stella et al. 2017). These empirical findings align with the tendency of children to acquire centrally connected concepts earlier (Hills et al. 2009; Cox and Haebig 2022).

### 2.10 Beyond Pairwise Networks: Hypergraphs as Cognitive Models

Hypergraphs extend the concept of traditional networks by allowing edges (called hyperedges) to connect more than two nodes simultaneously. Cognitive hypergraphs (introduced in Citraro, De Deyne, et al. 2023; Citraro, Warner‐Willich, et al. 2023) exploit a richer, more powerful mathematical representation compared to pairwise cognitive networks. In mathematical terms, an undirected hypergraph H is denoted as $H=\left(V,E\right)$ with V being a set of nodes or vertices and E being a set of hyperedges. Each hyperedge is a nonempty subset of V. A normal graph with edges containing exactly two nodes is called a 2‐hypergraph (Bretto 2013). In k‐regular hypergraphs, all hyperedges link exactly k different nodes. If k is not mentioned, then a hypergraph can feature hyperedges linking a variable amount of nodes. For example, if k=3 every hyperedge would link three distinct nodes (Bretto 2013).

**FIGURE 8:**
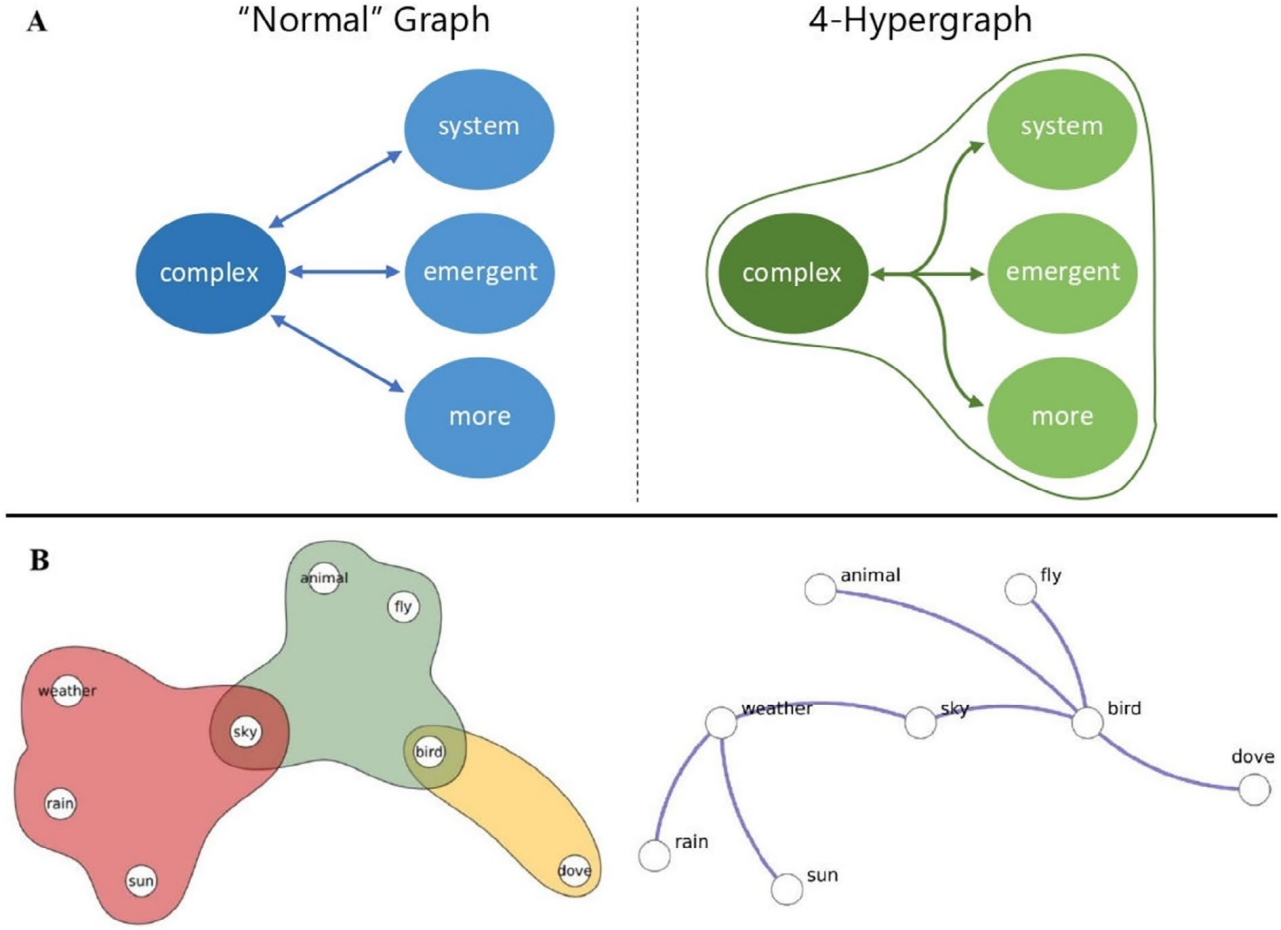
Examples of hypergraphs. Panel A: Responses from a free association task connected to the cue word as a “normal” graph (left) and a 4‐hypergraph (right). Left: The normal graph (pairwise network or 2‐hypergraph) consists of three edges connecting exactly two nodes each; that is, the cue word with each response separately. Right: The 4‐hypergraph consists of one edge connecting all four nodes; that is, the cue word with all responses combined. Panel B: Cognitive hypergraph (left) and pairwise network (right) for the free association data relative to {{“dove,” “bird”}, {“bird,” “animal,” “fly,” “sky”}, {“weather,” “sky,” “rain”, “sun”}}. Each set of responses is coded as a hyperedge with the first entry representing the cue, followed by the responses.

Hypergraphs are especially valuable in cognitive network science when dealing with situations where relationships naturally involve more than two entities. Consider free association tasks, where a cue word elicits multiple related responses. In these tasks, an individual reads a cue and then recalls at most three responses related to it (De Deyne et al. 2013). Mathematically, for a cue c and its responses $r_{1},r_{2}$ and $r_{3}$ the hyperedge between them can be represented as $e=\left\{c,r_{1},r_{2},r_{3}\right\}$. For instance, when prompted with “complex” a participant might think of “system,” “emergent,” and “more.” The responses from the free association task can be linked to the cue word as a “normal” graph with each response receiving its distinct edge (Figure 8A, left) or a 4‐hypergraph with one hyperedge connecting all four nodes *e* = {complex, system, emergent, more} (Figure 8A, right).

Representing this scenario as a pairwise network (or 2‐hypergraph) where edges exist only between the cue and individual nodes loses the information that all three responses were elicited together. De Deyne et al. (2013) showed that building pairwise free association networks, that is, featuring only links between the cue and its three responses, led to structural network measures that predicted a variety of lexical processing tasks better than alternative pairwise networks linking the cue and all responses with each other or considering only a connection between the cue and the first response.

The distinction between hypergraphs and pairwise networks is depicted in Figure 8B. The hypergraph (Figure 8B, left) encodes each set of responses as a hyperedge, preserving the full contextual grouping. In contrast, the pairwise network (Figure 8B, right) connects only two nodes at a time, resulting in a loss of higher‐order relational information.

Revisiting free association data, Citraro, De Deyne, et al. (2023); Citraro, Vitevitch, et al. (2023) introduced cognitive hypergraphs as a new idea at the interface of psychology and data science. Cognitive hypergraphs, where a hyperlink encapsulated both the cue and its responses, provided better models than pairwise networks in predicting psycholinguistic features like concreteness, a psycholinguistic norm indicating whether concepts are perceived as tangible or easy to picture (cf. Citraro, De Deyne, et al. 2023) or predicting normative early word learning across self‐reported norms and empirical norms in toddlers (cf. Citraro, Warner‐Willich, et al. 2023). These findings underline that in some specific tasks relative to lexical acquisition and access, considering weak relationships between targets can be insightful. Hypergraphs provide more flexible tools for capturing conceptual relationships between more than two elements at a time and the above pioneering results indicate cognitive hypergraphs as a promising direction for future research in cognitive network science.

## 3 Datasets and Software for Building Cognitive Networks

Computational scientists interested in analyzing cognitive networks should be aware of relevant datasets tailored to specific research interests. This section reviews both historical and more recent datasets, organized across different aspects of the mental lexicon. Python libraries (Python Software Foundation 2023) like *NetworkX* (Hagberg et al. 2008, https://networkx.org/, Accessed: 12/12/23) and *igraph* (Csardi and Nepusz 2006, https://igraph.org/, Accessed: 12/12/23) make it easy to import networks as edge lists while providing a wide variety of pre‐implemented network measures. Notice that *igraph* works also in R (R Core Team 2023) and Mathematica (Wolfram Research 2023). For R users, *MuxViz* (De Domenico et al. 2015) is a powerful tool for multiplex network analysis and visualization (De Domenico 2022). *SemNA* (Christensen and Kenett 2023) is another valuable R package that specializes in generating semantic networks out of verbal fluency data (Christensen and Kenett 2023).

### 3.1 Datasets and Software for Phonological Networks

Phonological networks map sound similarities between words (Vitevitch 2008). These similarities can be identified through shared phonemes, rhyme patterns or similar phonetic features (Vitevitch and Mullin 2021). Building phonological networks thus requires phonological transcriptions. A widely used approach is to adopt the International Phonetic Alphabet (IPA) and consider IPA transcriptions as strings of characters with each character representing a phoneme (Turnbull 2021). This is advisable due to potential problems with orthographic writing, where the same sequence of letters may represent different sounds in different words for non‐transparent languages (Aitchison 2012), for example, the English word “meet” differing from its IPA transcription /miːt/. IPA also contains characters not representing phonemes directly but still encoding for the duration of other phonemes. Therefore, it is most common to use phonetic word transcriptions for generating phonological networks (Vitevitch 2008), including or excluding non‐phoneme characters (Turnbull 2021). For “transparent languages,” where there is a consistent mapping between phonemes and letters, phonological networks can be built out of orthographic word transcriptions (Martinčić‐Ipšić et al. 2016). The “transparency” of these languages is constituted by these consistent rules for grapheme‐phoneme mapping, for example, learning that “ie” in German is pronounced /iː/. For non‐transparent languages, like English, ad‐hoc datasets are required to build phonological networks. The command WordData[] in Mathematica contains phonological transcriptions for over 35,000 English words. A more extensive resource is the IPA‐DICT project (https://github.com/open‐dict‐data/ipa‐dict, Accessed: 03/04/22), which gathers IPA transcriptions for 23 languages in multiple formats (including tsv, JSON and csv). Data scientists might also consider procedural pipelines, which read an orthographic input and apply sequences of transformations to get phonological transcriptions. One of these pipelines is the Epitran project (Mortensen et al. 2018), which comes as an easy‐to‐install Python library and supports over 90 languages.

As mentioned above, the local topology of words in a phonological network can correspond to facilitative or inhibitive patterns in word retrieval, confusability or short‐term retention (cf. Siew et al. 2019; Vitevitch and Mullin 2021). The R package *spreadR* (Siew 2019) simulates spreading activation over any single‐layer network structure, including phonological ones, to capture patterns of lexical retrieval like false alarm rates and inhibited spoken word recognition (Siew 2019). The package can be loaded via CRAN and gets as an input a network topology, the starting node, a retention rate (how much activation should not spread across links at each timestep), a dampening factor, the initial activation level and how many steps should be executed. The package can output the whole spreading dynamics, that is, sequences of activation levels across all timesteps for all nodes in the network. Extensively tested in phonological and semantic networks, *spreadR* represents a practical tool for harnessing how patterns of word recall or identification might emerge from the interplay of network structure and spreading dynamics (cf. Siew 2019).

### 3.2 Datasets and Software for Semantic Networks

Semantic networks codify meaning similarities or memory‐based relationships between concepts such as hierarchical relationships like “animal” and “mammal,” or associative links like “sun” and “day” (Kenett et al. 2018; Wulff et al. 2019; Valba et al. 2021). Dictionaries historically represented key data sources for building semantic networks (Miller 1995). However, Big Data and mega‐studies are quickly enriching the landscape of semantic data by providing vast automatic datasets (Steyvers and Tenenbaum 2005; Kennington and Schlangen 2015; Kumar et al. 2020). For example, Big Data about language from sources like social media have been used to track shifts in conceptual knowledge in response to real‐world events. Davis (2023) used Twitter data to study how the concept of COVID‐19 reshaped the meanings and relationships of related words in the semantic network, especially those tied to social interactions or emotions like fear. Tweets were collected mentioning specific topics and were investigated via a sliding window approach. This approach examines clusters of words within a fixed range (e.g., a window of five words on either side of a target word) to identify which words commonly appear in close proximity. By constructing semantic networks from this big language data, Davis (2023) observed that the semantic network shifted in such a way that emotionally and socially related words became more central in the network structure, acting as hubs for connecting diverse clusters of related concepts. This reflects their pivotal role in organizing concepts within the network, mirroring their heightened importance in societal discourse during the pandemic (Davis 2023). Similar analyses were performed by Semeraro et al. (2022) to identify keywords and shifts in network structure from over 5 K Italian news about COVID‐19 vaccines and discussed on Twitter.

While studies on Big Data about language as discussed above provide valuable insights into how the societal discourse around a topic can shift, in this paper we will instead focus on: (i) dictionary‐based data and (ii) behavioral or psychological data.

For dictionary‐based data, one of the largest corpora of semantic relationships is WordNet (Miller 1995; Fellbaum 1998), where words are organized according to sense and semantically related senses create *synsets*. For instance, “duck.n.01” refers to the animal with n.01 referring to a noun, whereas “duck.v.01” is a movement with v.01 referring to a verb. WordNet maps six semantic relationships:

1. **Synonyms**: Synonymous words that share an overlap in meaning in some contexts, for example, “forest” and “woods”;
2. **Hypernyms**: Hypernyms represent superordinate generalizations, that is, a concept representing a whole category of more specific concepts, like “bird” being a generalization of “dove”;
3. **Hyponyms**: Hyponyms are specifications of broader categories, that is, a concept being a specific instance of another concept representing a broader category, like “apple” being a specification of “fruit.”
4. **Antonyms**: Antonyms denote contraries, like “trust” and “distrust.”
5. **Meronyms**: Meronyms highlight a concept being a part of another, as seen in “wheel” being a part and thus a meronym of “car.”
6. **Holonyms**: Holonyms refer to concepts that include other parts within them. For example, “car” is a holonym for “engine,” “wheel,” and “seat” since these parts are components of a car. Thus, holonyms are the counterpart of meronyms, with holonyms describing the whole concept (“car”) and meronyms referring to a part of that whole (“wheel”).

In Python, WordNet 3.0 is available via the *wordnet* package in NLTK (the Natural Language ToolKit, see https://www.nltk.org/howto/wordnet.html, Accessed: 12/12/23). WordNet (Miller 1995) is also available in R and, natively, in Mathematica through the WordData[] command. Through the packages, one can access synsets and use them as edge lists (Steyvers and Tenenbaum 2005).

A pioneering study by Sigman and Cecchi (2002) examined the graph structure of WordNet to uncover how the English lexicon is globally organized. They found that WordNet behaves as a small‐world network, where a few highly connected hubs dominate the structure. These hubs often represent abstract, polysemous terms (i.e., terms with multiple meanings such as “head” being used for a human head, a letter head, or the head of a department). Polysemy plays a crucial role in compactly organizing the semantic network, facilitating efficient navigation through the network (Sigman and Cecchi 2002).

Whereas dictionary‐based data come from lexical resources mostly curated by expert linguists, psychological data include different types of semantic data coming from experimental setups. One type of data where semantic networks can be highly valuable tools is data on language acquisition. Semantic networks can offer insights into vocabulary development and the structure of lexical knowledge. They can reveal patterns in how words are learned, grouped, and interconnected by children acquiring a first language or adults learning a second or third language and can shed light on delayed language development (Agustín‐Llach 2023; Jiménez and Hills 2023; Stella et al. 2017). Research into early language acquisition reveals that children tend to group words by associative, categorical, and phonological links, which facilitates robust word learning. Simulations of vocabulary growth suggest that children have a tendency to acquire hubs earlier, a phenomenon known as preferential acquisition and increasingly studied with the help of cognitive network science (Borovsky and Peters 2019; Citraro, Warner‐Willich, et al. 2023; Stella et al. 2017; Stevyers and Tenenbaum 2005).

The semantic networks of typically developing children are characterized by high connectivity, clustering, and the presence of hub nodes. These structural characteristics support efficient navigation through the network and retrieval of words (Borovsky and Peters 2019; Stella et al. 2017). In contrast, late talkers (i.e., children with delayed language onset despite no other developmental delays or cognitive impairments) exhibit less complex lexical networks, with fewer connections and reduced clustering. These structural differences suggest difficulties in integrating new words into existing semantic structures and, thus, in establishing robust associative links between words (Jiménez and Hills 2023).

Another type of semantic data where constructing semantic networks can be highly informative is free association data. Free association is a well‐established method in cognitive science where participants are presented with a cue word and asked to respond with the first word that comes to mind (Nelson et al. 2004). Variations of the free association task may require participants to respond with the first 2–3 words that they associate with the cue (De Deyne et al. 2013; Nelson et al. 2000). The resulting cue‐response pairs reflect associative links in semantic memory. These associations are collected through experiments rather than extracted from sources like dictionaries, making them a valuable form of empirical data (De Deyne et al. 2013; Kenett et al. 2018; Wulff, Aeschbach, et al. 2022; Wulff, De Deyne, et al. 2022). Free associations can also be enriched with emotional data to obtain forma mentis networks, which are structural representations of how individuals or groups emotionally perceive and associate ideas in their memory (Ciringione et al. 2024). Additionally, they can help to identify individuals' perceptions of general topics (Aeschbach et al. 2024) or key target ideas, like maths anxiety (Ciringione et al. 2024).

As mentioned in Section 2.9, in the continued free association task a participant reads a cue (e.g., “pen”) and has to react with up to three responses that come to their mind as quickly as possible (e.g., “paper,” “writer,” “pencil”) (De Deyne et al. 2013, 2019). Free associations encode memory recalls, which are mostly semantic (Stella et al. 2017) but also multidimensional (De Deyne et al. 2019; Ciaglia et al. 2023): Free associations can come from phonological, orthographic, visual and other types of similarities between words. Currently, the Small World of Words (SWOW) project (De Deyne et al. 2019) is the largest repository of free association data across 17 languages (https://smallworldofwords.org/en/project/home, Accessed: 12/12/23). Unlike traditional dictionaries or thesauri, SWOW utilizes word associations to uncover the meanings and centrality of words in the human mind. The SWOW project involves presenting participants with words and prompting them to provide spontaneous associations, that is, to perform the continued free association task. SWOW represents a rich, large‐scale dataset: Currently, SWOW counts over 700,000 links between 40,000 English words and it keeps growing (De Deyne et al. 2019). Free associations powered a wide variety of applications in the last few years, including the interplay between semantic memory and concepts related to beauty and wellbeing (Kenett et al. 2021), or suicide ideation (Teixeira et al. 2021), or personality traits (Kenett et al. 2018). Free association networks using SWOW enabled researchers in exploring also the intricacies of word relationships, including complex dimensions like individual differences (Wulff, Aeschbach, et al. 2022), creativity levels (Stella and Kenett 2019), mental well‐being (Fatima et al. 2021) and cognitive processes across aging (Dubossarsky et al. 2017; Wulff et al. 2019; Wulff, De Deyne, et al. 2022). SWOW thus represents a promising dataset for next‐generation models of cognition and human behavior.

Other notable mentions of free association datasets include the University of South Florida Free Association norms including 70,000 links between 10,000 American English words (Nelson et al. 2004)—and the Edinburgh Associative Thesaurus containing 325,000 links between 23,000 British English words (Wilson and Kiss 1988). For an overview of key studies using free association data to build cognitive networks, see the Further Reading section at the end of this paper.

Behavioral semantic data can come also as fluency lists (Rastelli et al. 2022; Fatima et al. 2021; Siew and Guru 2023), that is, ordered sequences of words relative to a given category (as already mentioned in Section 2.8). For instance, in a category task, individuals produce fluency lists of as many words as possible from a given category within a time limit of 2 min (Goñi et al. 2011). The number of responses measures the so‐called linguistic fluency and it has been used historically as a psychometric measurement of language skills (see also Zemla et al. 2020). However, fluency lists should be seen as the outcome of mental search and retrieval processes of a highly complex nature and potentially exploiting some network aspects of the mental lexicon (Hills and Kenett 2021). Cognitive networks built from fluency lists can go beyond simple fluency measures and unveil the structure among consecutive responses. How to assess the cognitive network structure embedded but not immediately apparent in fluency lists? There is no unique answer to this research question, however most studies on the topic tend to follow mostly two psychometric approaches: (i) consider fluency lists as ordered sequences of cognitive data where subsequent or co‐occurring responses share stronger semantic similarities than concepts further apart in the sequence, or (ii) consider fluency lists as observed data coming from a latent cognitive network structure, which can be inferred via mathematical modeling.

Historically, the first approach was the first one to appear in the literature (cf. Goñi et al. 2011), inspired by word co‐occurrences (Amancio 2015). Goñi et al. (2011) introduced a simple yet elegant co‐occurrence Binomial filtering for tracing which concepts tended to co‐occur more than randomly expected in sets of fluency lists (Goñi et al. 2011). Their approach was thus psychometric in that they considered a null model and filtered against spurious co‐occurrences. When conducting the category fluency task on the topic “animals,” the resulting fluency networks were typically characterized such that animals sharing several semantic features (such as appearance or habitat) tended to be more tightly connected to each other than to other animals in the network.

Fluency data can also be considered as the outcome of random walks over latent cognitive structures. Hence, making semantic networks based on fluency data can be considered the outcome of an inference problem. This means that building such networks involves making mathematical inferences based on the fact that the observed fluency data is the outcome of a stochastic or random process, one observation among many possible ones. A tool that can be utilized for building fluency networks in this way is the package *SNAFU* in Python (Zemla et al. 2020, https://github.com/AusterweilLab/snafu‐py, Accessed: 04/12/23). *SNAFU* stands for *Semantic Network and Fluency Utility* and helps, among other functions, with counting clusters, perseverations, and calculating word frequency (Zemla et al. 2020). This reconstruction approach created cognitive networks whose features automatically classified people affected by early Alzheimer's Disease (against healthy controls) with an accuracy of over 92% and an F1 score of 0.88 (Zemla and Austerweil 2019). These datasets and methodologies all open the way to exciting data‐informed explorations of mental search processes.

**FIGURE 9:**
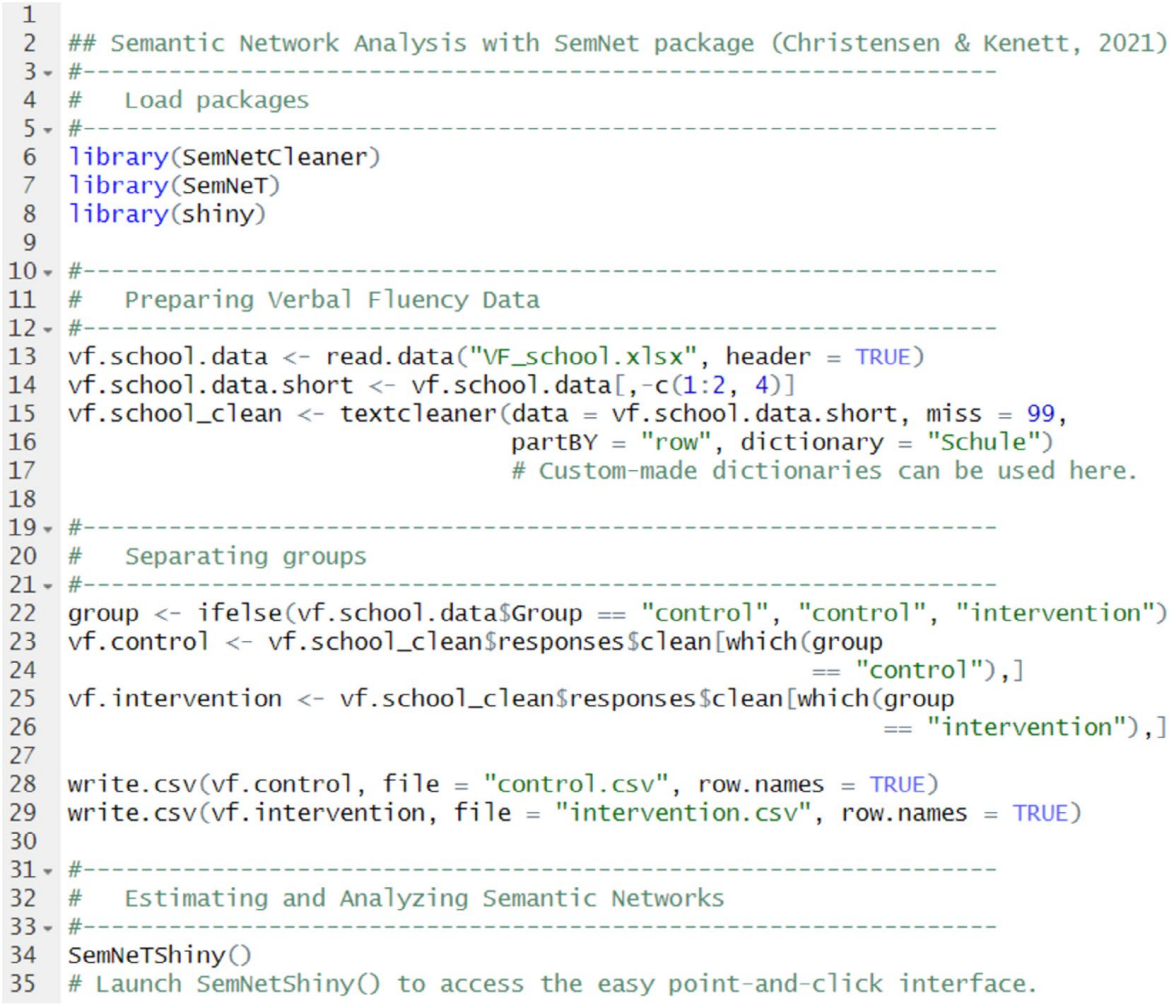
Example code used within the *SemNA* package in R (Christensen and Kenett 2023). The package has an integrated module for preprocessing and cleaning raw fluency data. Dictionaries for British and American English are available and can be extended with custom‐made dictionaries (see *dictionary =* “NAME OF DICTIONARY”). Lastly, by launching SemNetShiny(), one is forwarded to an easily accessible point‐and‐click interface where semantic network analyses are conducted.

An intermediate (set of) approach(es) between network inference and co‐occurrence analysis is the *SemNA* package (Christensen and Kenett 2023), which transforms fluency data in cognitive networks and provides accessible tools for network analysis with a comprehensive point‐and‐click interface (Christensen and Kenett 2023). This package incorporates several R modules, including SemNetDictionaries, SemNetCleaner, and SemNeT. These modules play integral roles in preprocessing, data cleaning, and semantic network generation. Notably, SemNA facilitates the generation of four distinct networks, each grounded in diverse network theories (Christensen and Kenett 2023). Figure 9 portrays a snippet of example code in R for using the SemNet package. In a first step, the verbal fluency data is prepared by using the *textcleaner* function for automatic spelling correction, identification and exclusion of inappropriate words. Next, group data is divided in order to generate semantic networks and calculate differences for each group, including statistical testing via *t*‐tests and bootstrapping. Finally, the SemNetShiny() command opens an easily accessible point‐and‐click interface where statistical and graphical semantic network analyses are conducted.

Recent studies used fluency networks (built in either way) that can unveil crucial differences in the way groups of individuals with different altered cognitive states (Rastelli et al. 2022), age (Cosgrove et al. 2021), education levels (Denervaud et al. 2021), or domain knowledge proficiency (Siew and Guru 2023) structured their semantic memories. These recent and intriguing results underline how giving structure to fluency data can unveil interesting cognitive and psychological patterns.

### 3.3 Datasets and Software for Syntactic Networks

Syntactic networks explicate grammatical and meaning relationships between words in sentences (Ferrer i Cancho et al. 2004). For instance, “Love is weakness” syntactically specifies a property (“weakness”) of a noun, “love,” through a verb. This sentence might thus be represented as the undirected link (*love, weakness*). Extracting syntactic relationships is known as *parsing*. In the human mind, this complex task involves a variety of complex phenomena taking place in sequences that are partly unknown (cf. Dóczi 2019) but include: breaking down sentences into their grammatical components, identifying the relationships between words, and understanding the overall meaning conveyed by the arrangement of linguistic elements. In the context of syntactic analysis for research, the task of parsing has long been left to human coding (Carley 1993), which refers to the manual process of annotating and categorizing linguistic elements and relationships between them within a given text. The time‐consuming process of human coding has been revolutionized with the advent of soft computing models like the Stanford Universal Parser (Dozat et al. 2017) or spaCy's library (https://spacy.io/usage/linguistic‐features#dependency‐parse, Accessed: 12/12/23), which implement automatic syntactic parsing via artificial intelligence (AI).

Before automatic syntactic parsing, word co‐occurrences have been a computationally advantageous proxy for extracting local syntactic relationships from text (Amancio 2015). Word‐word co‐occurrences identify which words in sentences tend to appear as adjacent or separated by l words. Co‐occurrences can be discarded if below a threshold (Amancio 2015; Teixeira et al. 2021) or filtered against random expectation (Quispe et al. 2021). For a pedagogic tutorial about how to build word co‐occurrence networks in Python, see the Semantic Brand Score Package (Colladon 2018, https://github.com/iandreafc/semanticbrandscore‐demo, Accessed: 12/12/23), which was used to monitor brand perceptions online via co‐occurrences in users' reviews (Colladon 2018). Word co‐occurrences in adults' child‐directed speech contributed to predicting early word learning (Stella et al. 2017) and differed in structure from adults' general speech (Cox and Haebig 2022).

While more computationally costly, automatic syntactic parsing captures non‐local syntactic dependencies that would be lost with co‐occurrences. Consider the sentence “Climate change is such a terrible, disastrous, problematic issue” in which “change” and “issue” are syntactically related but separated by three adjectives. Thus, co‐occurrence networks with l<3 would miss this syntactic relation due to the number of intervening specifications.

Capturing all syntactic relationships in a text means automating content mapping (Carley 1993) and operationalizing the automatic constructions of sets of words all referring to a given target word *x*, that is, the automatic construction of the semantic frame for *x* (Fillmore and Baker 2012). Both frame semantics and content mapping are frameworks capturing stances, frames, mindsets or perceptions, which in general terms all rely on specific ways of associating and combining ideas in communicative intentions (Stella 2022a). According to the theory of frame semantics (Fillmore and Baker 2012), reconstructing the semantic frame of a word is enough to understand its meaning in a given text. Differently put, frame semantics indicates that the meaning of a word can be inferred from other words that “keep it company,” that is, syntactically related concepts.

Syntactic networks can, thus, reconstruct a set of stances, frames and even, to a limited extent, a fragment of the mindset of a text author, as shown with recent work about *textual forma mentis* networks (TFMNs, forma mentis meaning “mindset” in Latin) (Stella 2020a). Differently from other NLP (Teixeira et al. 2021) or co‐occurrence (Quispe et al. 2021) proxies to syntactic links, textual forma mentis networks enrich AI‐detected syntactic links with synonym relationships and affective norms to better represent the cognitive‐affective content of texts. Importantly, TFMNs use an AI to perform syntactic parsing and then, on the resulting tree of syntactic dependencies, TFMNs link only pairs of words possessing meaning and at a distance lower than a threshold *t* on the syntactic tree, rather than in the original sentence. This difference with word co‐occurrence networks crucially makes it possible for TFMNs to capture syntactic relationships between words not adjacent or close in a sentence and yet syntactically related.

**FIGURE 10:**
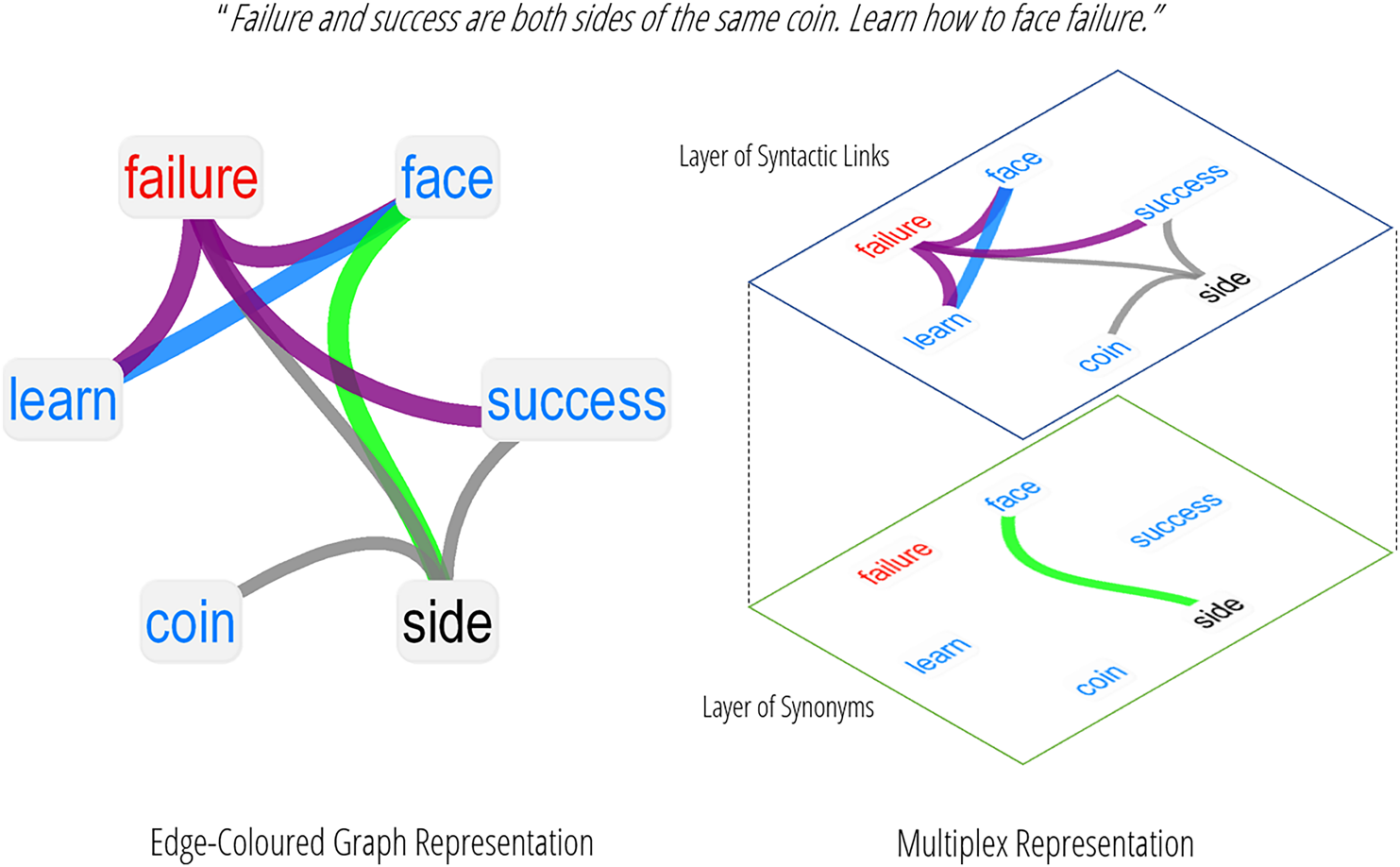
A textual forma mentis network (TFMN) for the text “Failure and success are both sides of the same coin. Learn how to face failure.” Words are highlighted in red (black, cyan) if rated as negative (neutral, positive) in the Emotional Lexicon. Syntactic links are colored as the words at their endpoints. Synonyms are in green and come from WordNet. TFMNs can be visualized as either edge‐colored graphs (left) or multilayer networks (right). The figure was constructed via the EmoAtlas library in Python (Semeraro et al. 2025).

Figure 10 reports a toy example of a TFMN: “failure” by itself is rated negatively by humans (cf. The Emotion Lexicon by Mohammad and Turney 2013). However, in the sentences “Failure and success are both sides of the same coin. Learn how to face failure.” (see Figure 10) it is syntactically linked with mostly positive concepts such as “learn” and “success.” The TFMN approach thus unveils that “failure” in that sentence is framed along a positive connotation (i.e., overcoming failure). Textual forma mentis networks can be built through the EmoAtlas library (Semeraro et al. 2025) in Python (https://github.com/MassimoStel/emoatlas, Last Accessed: 07/07/25) and they can be used to identify stances as well as emotional profiles of different texts. The EmoAtlas package can either be used in Google Colab or can be downloaded from GitHub (https://github.com/MassimoStel/emoatlas, Last Accessed: 07/07/25) and, once imported, enables the extraction of TFMN edge lists and the visualization of specific semantic frames (like the one in Figure 10, left) with the aid of colors and hierarchical edge bundling (i.e., a heuristic placing nodes on a circle and putting those sharing links closer). This granularity has been used in studies to highlight crucial differences in the ways COVID‐19 vaccines were framed by mainstream media and alternative news outlets (Semeraro et al. 2022).

Another compelling option for generating text for syntactic analysis is the Woseco task, short for “word‐sentence‐construction” (Haim, Lai, et al. 2024; Haim, Fischer, et al. 2024). Originating as a teaching method developed by Haim and Aschauer (2022), the Woseco task aims to enhance the verbal creativity of pupils within the context of a specific school subject. In this task, an instructor provides a technical term from a broad category. Task participants then have to construct a factually correct sentence that not only includes the provided category but also incorporates a second technical term—of their choice—with which they will build another sentence related to the category. The process is iterated several times (Haim and Aschauer 2022). Leveraging automated analysis tools like spaCy in Python (https://spacy.io/, Accessed: 24/11/23), it becomes possible to extract target words (typically nouns) from these sentences, building a network of syntactic dependencies that differs from TFMNs. This integration of Woseco into syntactic analysis not only provides a valuable resource for understanding language dynamics but can also enrich the exploration of verbal creativity in educational contexts (Haim, Lai, et al. 2024, Haim, Fischer, et al. 2024).

Syntactic parsing can also be enhanced by entity recognition. Entity recognition involves identifying and classifying specific entities, such as names, locations, or other meaningful elements, within a given text or context. Enhancing syntactic parsing through entity recognition allows for a more detailed understanding of the relationships and roles of identified entities in the analyzed text. The recent *netts* library (NETworks of Transcript Semantics, https://pypi.org/project/netts/, Accessed: 04/12/23) can disambiguate entities in texts, for example, “Exeter – the University” versus “Exeter – the city”, and draw syntactic relationships between clusters of them, such as “Exeter → is a red brick → University” (Nettekoven et al. 2023). Relying on CoreNLP (Manning et al. 2014) and its recurrent neural network models (https://stanfordnlp.github.io/CoreNLP/, Accessed: 04/12/23), *netts* is currently available in Python. When analyzing transcripts produced by people affected by psychosis, *netts* unveiled key differences in network structure compared to healthy controls. This represents evidence that syntactic networks can enable a deeper understanding of ways of thinking as encoded also in texts written by clinical populations. The sentence structure and grammar of such clinical texts can provide insight into the thought processes of clinical populations which can be better understood through cognitive network science (Lydon‐Staley et al. 2019; Parola et al. 2023).

### 3.4 Software for Network Visualization

In analyzing the above‐mentioned phonological, semantic, and syntactic networks, visualization plays an important role in giving form to the network and allowing a more intuitive understanding of complex relationships within the network. Here, we briefly introduce *igraph* (Csardi and Nepusz 2006), *NetworkX* (Hagberg et al. 2008), *ggraph* (Pedersen 2022), and *tidygraph* (Pedersen 2020), four useful toolboxes for creating customizable network plots. Since they can be applied for both phonological, semantic, and syntactic networks alike, we discuss them in this separate section to emphasize how they support cognitive network research across different types of networks.

For users of Python, *igraph* (Csardi and Nepusz 2006) and *NetworkX* (Hagberg et al. 2008) are practical visualization tools. Both libraries support easy import of networks as edge lists, adjacency matrices, and a variety of built‐in network measures, such as clustering coefficients, centrality measures, and path lengths. The *igraph* package (Csardi and Nepusz 2006), which is also available in R, performs especially well on large networks and offers both 2D and 3D visualization options (https://igraph.org, Accessed: 14/11/24). Meanwhile, *NetworkX* (Hagberg et al. 2008) is particularly user‐friendly for moderately sized networks and is practical for exploratory analysis and interactive plots (https://networkx.org, Accessed: 14/11/24).

**FIGURE 11:**
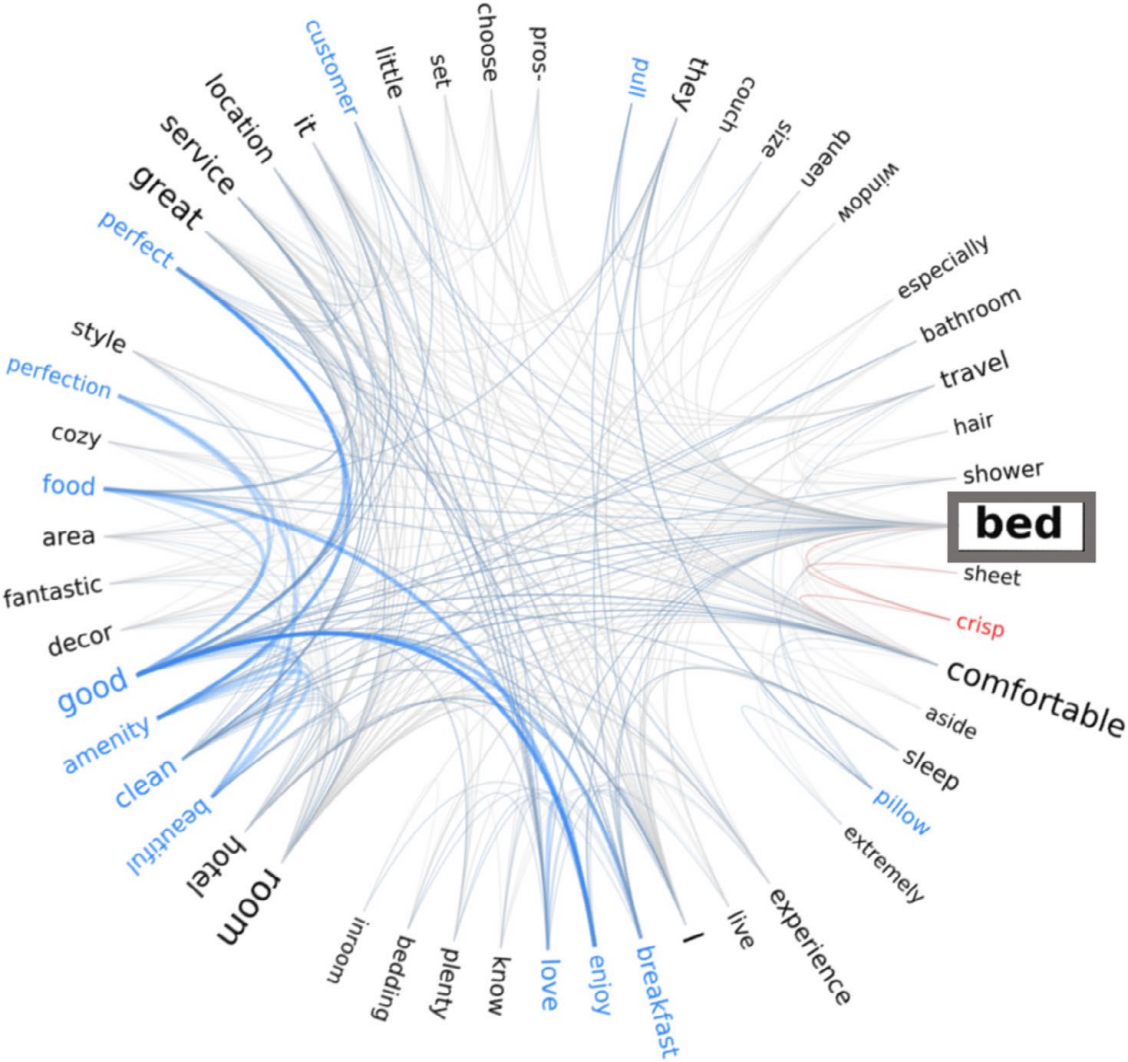
Hierarchical edge bundling layout for the associates of “bed” in a corpus of positive hotel reviews. The bundling, obtained via *igraph* (Csardi and Nepusz 2006) and *EmoAtlas* (Semeraro et al. 2025), organizes associations along a circular layout and bundles edges together by putting words more connected with each other closer on the circular layout. As in textual forma mentis networks, nodes are colored according to their valence (cyan for positive, red for negative, black for neutral) and links are colored according to the valences of its connected words (e.g., links between positive words are thicker and cyan).

Both *ggraph* (Pedersen 2022) and *tidygraph* (Pedersen 2020) are part of the tidyverse ecosystem in R. The *ggraph* package is designed for visualizing complex network structures and works seamlessly with *igraph* (https://igraph.org/, Accessed: 14/11/24). Layout options include force‐directed and circular arrangements, which highlight clusters or hierarchical relationships. An example is reported in Figure 11, which displays syntactic associations to the word “bed” in a corpus of positive hotel reviews, as gathered in Semeraro et al. (2025). The graphical layout, obtained in Python, organizes associations along a circular layout, bundling edges so that words more interconnected with each other are closer in the layout. This visualization was obtained in Python, via *igraph* (Csardi and Nepusz 2006) and a user‐friendly package called *EmoAtlas* (Semeraro et al. 2025), available in Google Colab (https://github.com/MassimoStel/emoatlas, Last Accessed 07/07/2025) and creating textual forma mentis networks from text (Semeraro et al. 2025; Stella 2020a). A step‐by‐step tutorial for the EmoAtlas library can be found in Section 3.5.

Users can also choose tree views, which are useful for displaying nested structures (https://ggraph.data‐imaginist.com/index.html, Accessed 14/11/24). In contrast, *tidygraph* (Pedersen 2020) focuses on data manipulation within network structures, allowing researchers to prepare and customize data easily. The *tidygraph* package enables filtering, transforming, and arranging network elements. This can be essential for larger networks where certain nodes or edges require highlighting or adjustment (Pedersen 2020, https://tidygraph.data‐imaginist.com, Accessed 14/11/24).

### 3.5 Short Tutorial: EmoAtlas Walkthrough

This short walkthrough introduces the open‐access package EmoAtlas (Semeraro et al. 2025), which was used in the present paper to generate story‐based network visualizations (see Visual Abstract, Figures 10 and 11). The goal of this walkthrough is to help readers understand how to use EmoAtlas for analyzing their own text data. So far, EmoAtlas has been tested in English and Italian, with work underway to expand to additional languages.

#### 3.5.1 Step 1: Install and Import the Required Libraries

**FIGURE 12:**
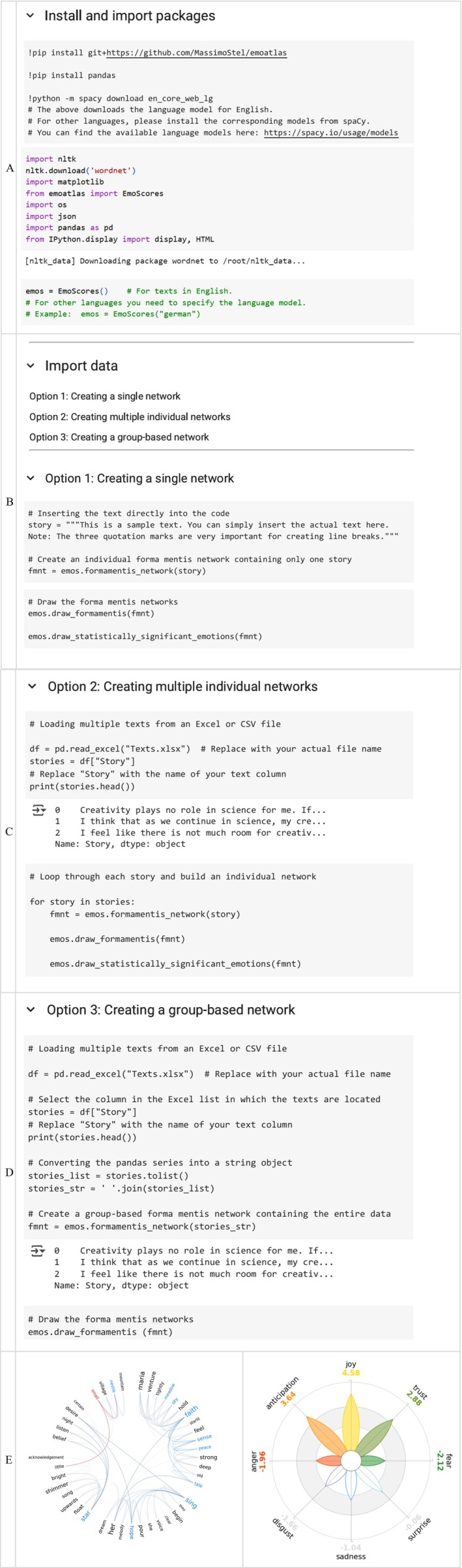
Code snippets leading through the EmoAtlas package in Python (Semeraro et al. 2025). Panel A: Installing and importing the necessary packages. Panel B: Creating a network from a single text by copying the text directly into the code. Panel C: Creating multiple individual networks from a dataset using a loop. Panel D: Creating a group‐based network from multiple texts. Panel E: Example output for a forma mentis network (left) and emotion flower (right).

Figure 12, panel A shows a basic code snippet to get started. This includes installing EmoAtlas and related packages (such as spaCy and pandas) in a Python environment (here we used Google Colab). It also shows how to download the appropriate spaCy language model (see for more models https://spacy.io/usage/models). When setting up the EmoScores object, users specify the language of the text. For English, the argument after *emos = EmoScores ()* can be left empty. For other languages, the appropriate model needs to be specified using quotation marks (e.g., “german”).

#### 3.5.2 Step 2: Load and Analyze Your Text Data

There are different ways to load and analyze the data, depending on whether it is a single text or a group of texts.

##### 3.5.2.1 Option 1: Analyze a Single Text to Create a Single Network

For users who want to analyze one specific text, the simplest way is to copy the text directly into the code. See Figure 12B for a basic code example. Note that triple quotation marks are needed to allow line breaks in the inserted text.

##### 3.5.2.2 Option 2: Analyze Multiple Texts to Create Individual Networks

In case of multiple texts, such as short stories from several participants, users can load them from an Excel or CSV file. By looping through each story in the indicated column where the stories are located, separate networks are created for each one. This is ideal when each text shall be analyzed and represented as a network separately. In the setup shown in Figure 12C, each text is treated individually and a forma mentis network and emotion flower are created for every entry in the specified “Story” column.

##### 3.5.2.3 Option 3: Analyze Multiple Texts to Create a Group‐Based Network

If a researcher is interested in the overall emotional and conceptual structure across a whole group, the texts can be combined into one string to generate a single group‐based network. Figure 12D shows how to merge the texts together to generate one combined forma mentis network for the group and capture the emotional patterns in a group‐based emotion flower.

#### 3.5.3 Step 3: Understanding the Output

The EmoAtlas package produces two key visualizations: a forma mentis network and an emotion flower (see Figure 12, panel E). The forma mentis network (panel E left) captures how terms within a text or a collection of texts are linked with each other. Nodes represent words and vary in size depending on how frequently they appear (bigger font means higher frequency). Lines (edges) between words indicate that they are associated with each other, while the thickness of the line indicates how strongly the terms are associated. The color of each word reflects its emotional tone: blue for positive, red for negative, and black for neutral. The edges are also color‐coded based on whether they connect two positive (blue), two negative (red), or one positive and one negative word (purple) (Semeraro et al. 2025; Haim, Fischer, et al. 2024).

The emotion flower (see Figure 12, panel E right) illustrates the emotional richness of the text based on eight core emotions: joy, trust, fear, surprise, sadness, disgust, anger, and anticipation. Each petal represents one emotion, and its length reflects how strongly that emotion is present. The values shown in the emotion flower are *z*‐scores, which indicate how often each emotion appears in the text compared to what would be expected by chance. This is done by comparing the emotional content of the text to a null model, which simulates random word use based on a reference lexicon (EmoLex). Positive *z*‐scores mean an emotion is significantly more present than expected; negative values mean it is less present. Petals that go beyond the gray threshold area (±1.96) are statistically significant and highlighted in color, showing emotions that are meaningfully over‐ or underrepresented in the text. For example, a long yellow petal for joy suggests this emotion is strongly present, while a short red petal with a negative score indicates that anger is notably absent in a given text (Semeraro et al. 2025; Haim, Fischer, et al. 2024).

## 4 Limitations and Future Work

Cognitive network science is deeply rooted in the mental lexicon metaphor and in associative knowledge modeling. Cognitive networks are powerful tools for revealing underlying structures in data that may appear unorganized or lack explicit structure, such as raw texts, recall data, or responses from lexical tasks. By mapping these data onto a network, cognitive networks unveil patterns in the organization, acquisition and framing of conceptual representations that might remain hidden with other, unstructured modeling frameworks. For example, cognitive networks outperform latent semantic spaces in explaining similarity ratings (Kenett et al. 2017). However, there are also several limitations to the field that should be acknowledged, discussed, and potentially relaxed via future research.

Firstly, whereas the mental lexicon is highly dynamic (Aitchison 2012; Zock 2020), most cognitive network models currently assume the mental lexicon to be static, that is, the layout of conceptual associations does not change over time. However, pioneering studies with free associations (Dubossarsky et al. 2017; Wulff et al. 2019; Wulff, De Deyne, et al. 2022) and fluency data (Cosgrove et al. 2021) have shown that indeed semantic memory in the lexicon changes over time. Alterations in the mental lexicon can be due to various factors such as experience, learning and exposure to new information (Wulff, Aeschbach, et al. 2022). As individuals encounter new words and concepts, their mental lexicon dynamically adapts, incorporating new associations to reflect evolving semantic structures and relationships (Storkel 2002; Beckage et al. 2011). Next‐generation cognitive network models should thus be able to account for time‐evolving conceptual associations. Relevant applications could be the investigation of treatment effects (Veltri 2023) or other aspects of aging (Wulff, Aeschbach, et al. 2022). A promising route would be the adoption of recent frameworks like stream graphs, where individual links can appear, persist and vanish over time, altering node centrality and network dynamics (cf. Citraro et al. 2022).

Secondly, the mental lexicon is multi‐faceted (Zock 2020) and cognitive networks must encompass multiple aspects of knowledge at once. Multiplex lexical networks already account for multiple types of conceptual links (Stella et al. 2017, 2018). The combination of these various conceptual links highlights phenomena invisible to single‐layer networks, for example, distance‐based mechanisms in predicting picture naming performance in people with aphasia (Castro and Stella 2019). However, these models are limited to mapping only the same set of nodes across layers, for example, only words, whereas the mental lexicon might include elements like phonemes or sentences (Aitchison 2012), or even specific regions of the human brain activated by priming (Zaharchuk and Karuza 2021). Multilayer networks, that is, generalizations of edge‐colored graphs where different sets of nodes can be linked across layers (De Domenico 2022), represent a valuable future direction for quantitative interpretations of psycholinguistic data. With some pioneering studies investigating the multilayer structure of language (Martinčić‐Ipšić et al. 2016), future research should deploy these models for interpreting more psychological data.

Thirdly, whereas most studies work at group level (Castro and Siew 2020), for example, comparing creativity between scientists and artists (Merseal et al. 2023) or across different age groups (Cosgrove et al. 2023), cognitive network models of individuals' mental lexica might provide a major modeling advancement. Cognitive networks generated on the level of individuals might be especially beneficial in digital ecosystems where user‐level data is already available (Mokryn and Ben‐Shoshan 2021), thus potentially mapping how individuals perceive, link and express ideas through online communicative intentions. The SWOW database (Small World of Words, https://smallworldofwords.org/en/project/home, Accessed: 24/11/23) recently collected free associations at individual level (Wulff, Aeschbach, et al. 2022; Wulff, De Deyne, et al. 2022). This enables a granularity that is crucial for pushing cognitive network science towards embracing individual assessment and variability, making cognitive networks interesting objects of investigations even for researchers already familiar with complex networks, for example, from network psychometrics (Golino and Epskamp 2017; Golino et al. 2020).

Fourthly, cognitive networks are not always the best model for explaining cognitive phenomena. For example, Kumar et al. (2020) found that word embeddings obtained from the word2vec method provided results slightly better than network distances in explaining priming data (Kumar et al. 2020). However, word2vec and cognitive networks captured different underlying structures, suggesting that combining model results into a complementary perspective could be more informative. Word embeddings quantify semantic similarity based on contextual co‐occurrence, while cognitive networks model the structure of associative knowledge. When used together, they may cross‐validate and enrich each other's findings (Zock 2020). Analyzing narratives provided by clinical populations, the word embedding approaches of Litovsky et al. (2022) and of Parola et al. (2023) both identify abnormal patterns analogous to ones detected by syntactic networks in Nettekoven et al. (2023) or described in Lydon‐Staley et al. (2019). Next‐generation models should use vectorial and network aspects of the mental lexicon in synergy. A promising example is DASentimental (Fatima et al. 2021), which combines semantic network distance and word embeddings to predict levels of anxiety, depression and stress from combinations of emotional words. Using a cognitive network embedding technique, a multi‐perceptron model was trained that demonstrated an ability to predict emotional distress levels in individuals with a performance comparable to that of human raters (https://github.com/asrafaiz7/DASentimental, Accessed: 11/11/2024).

Last but not least, cognitive networks primarily examine the structure of conceptual connections, yet the mental lexicon includes a distributional dimension, representing words as numerical vectors (Kumar 2021; Litovsky et al. 2022). Addressing the challenge of reconciling these two aspects, the recent Feature Rich Multiplex Lexical Network (FERMULEX) framework integrates both the vector and network elements of associative knowledge. This approach reveals patterns in early sentence production by toddlers that remain unnoticed by models solely focused on either network or vector representations (Citraro et al. 2022). While FERMULEX considers only language‐based features (e.g., frequency, length, polysemy), future models should account also for word‐level task‐based features such as concreteness (Citraro, De Deyne, et al. 2023), specificity or how context‐specific concepts are perceived (Bolognesi and Caselli 2023) or even latency—how easily concepts can be recalled or identified (Kumar et al. 2020; Litovsky et al. 2022). Extending network measures to feature‐rich networks might also provide additional insights over the vector nature of the mental lexicon. As an idealized system, the mental lexicon influences knowledge processing in ways mediated by vector similarities in some phenomena and by network structure in others (cf. Aitchison 2012; Zock 2020; Kovács et al. 2021; Hills and Kenett 2021; Citraro et al. 2022). This underlines the need for computational investigations of such complex cognitive systems through a *multiverse* approach (Parola et al. 2023), where multiple models are compared, combined and reconciled.

## 5 Conclusions

This paper has explored recent advancements in cognitive networks, emphasizing their role as models for understanding human cognition and behavior. Their applications range from deciphering cognitive processes across visual, auditory, and semantic tasks in diverse populations to predicting cognitive development, decline, and performance in both clinical and healthy contexts. Additionally, cognitive networks contribute to the reconstruction of semantic framing within texts and media. As a useful tool for modeling human behavior, the field of cognitive networks stands poised for growth, guided by meticulous statistical modeling, collaborative synergy with other interpretable frameworks, and the availability of rich datasets and powerful software packages spanning various tasks and contexts.

## Author Contributions

**Edith Haim:** writing – review and editing (equal). **Massimo Stella:** supervision (lead), writing – original draft (lead), writing – review and editing (equal).

## Conflicts of Interest

The authors declare no conflicts of interest.

## Related WIREs Articles

Models of spoken‐word recognition

Psychology of knowledge representation

Cognitive science as complexity science

## Data Availability

Data sharing is not applicable to this article as no new data were created or analyzed in this study.
